# Natural variation modifies centromere-proximal meiotic crossover frequency and segregation distortion in *Arabidopsis thaliana*

**DOI:** 10.1093/genetics/iyaf264

**Published:** 2025-12-08

**Authors:** Nicola Gorringe, Stephanie Topp, Robin Burns, Sota Yamaguchi, Fernando A Rabanal, Joiselle B Fernandes, Detlef Weigel, Tetsuji Kakutani, Matthew Naish, Ian R Henderson

**Affiliations:** Department of Plant Sciences, University of Cambridge, Downing Street, Cambridge, Cambridgeshire CB2 3EA, United Kingdom; Department of Plant Sciences, University of Cambridge, Downing Street, Cambridge, Cambridgeshire CB2 3EA, United Kingdom; Department of Plant Sciences, University of Cambridge, Downing Street, Cambridge, Cambridgeshire CB2 3EA, United Kingdom; Department of Plant Sciences, University of Cambridge, Downing Street, Cambridge, Cambridgeshire CB2 3EA, United Kingdom; Department of Biological Sciences, Graduate School of Science, University of Tokyo, Tokyo, Kantō 153-8902, Japan; Department of Molecular Biology, Max Planck Institute for Biology Tübingen, Tübingen, Baden-Württemberg D-72076, Germany; Department of Plant Sciences, University of Cambridge, Downing Street, Cambridge, Cambridgeshire CB2 3EA, United Kingdom; Department of Molecular Biology, Max Planck Institute for Biology Tübingen, Tübingen, Baden-Württemberg D-72076, Germany; Department of Biological Sciences, Graduate School of Science, University of Tokyo, Tokyo, Kantō 153-8902, Japan; Department of Plant Sciences, University of Cambridge, Downing Street, Cambridge, Cambridgeshire CB2 3EA, United Kingdom; Department of Plant Sciences, University of Cambridge, Downing Street, Cambridge, Cambridgeshire CB2 3EA, United Kingdom

**Keywords:** centromere, meiosis, recombination, segregation distortion, *Arabidopsis*, natural variation, CENH3, DNA methylation, satellite repeats

## Abstract

Eukaryotic centromeres mediate chromosome segregation during cell division. Plant centromeres are loaded with CENH3-variant nucleosomes, which direct kinetochore formation and spindle-microtubule interaction. Centromeres are frequently composed of megabase-scale satellite repeat arrays, or retrotransposon nests. In monocentric genomes, such as the model plant *Arabidopsis thaliana*, pericentromeric heterochromatin surrounds the CENH3-occupied satellite arrays. A zone of suppressed meiotic crossover recombination contains the centromere and extends into the pericentromeres. Here, we explore how natural variation in Arabidopsis influences centromere-proximal crossover frequency and segregation distortion when centromeres are heterozygous. We used fluorescent crossover reporters to quantify the effect of genetic variation on centromere-proximal recombination in 12 F_1_ hybrids between the reference strain Col-0 and nonreference accessions that captured Eurasian and relict diversity, and in total, we measured 3,037,802 meioses. The majority of the F_1_ hybrids (49 of 60) had significantly higher or lower centromere-proximal crossover frequency than inbreds. We relate hybrid crossover frequencies to patterns of nucleotide diversity and centromeric structural variation, and in a subset of 7 accessions, to epigenetic patterns of CENH3 enrichment and DNA methylation. Using linear modeling, we observed that chromosome and accession, and their interaction, together explained 85% of variation in crossover frequency, consistent with *cis*- and *trans*-acting modifying effects. The fluorescent reporters also allow segregation distortion through meiosis to be quantified between hybrids and inbreds. We observed a minority of hybrids (18 of 60) with distorted segregation through meiosis compared to inbreds, which occurred with or without a simultaneous change to centromere-proximal crossover frequency. Linear modeling revealed that 56% of variation in segregation distortion is explained by chromosome and accession, but with a stronger effect of accession compared to crossover frequency. We discuss how Arabidopsis centromeric structural heterozygosity may modify recombination and cause segregation distortion through meiosis.

## Introduction

Meiosis is a specialized eukaryotic cell division, where a single round of DNA replication is coupled to 2 rounds of chromosome segregation, which generates a tetrad of spores, each with half the number of chromosomes (Villeneuve and Hillers 2001; Mercier et al. 2015). During meiosis, interhomolog recombination is initiated during prophase I, via the formation of DNA double-strand breaks (DSBs) along the length of chromosomes by SPO11 complexes (Arter and Keeney 2024). Meiotic DSB formation takes place in the context of a proteinaceous axis—a multiprotein scaffold that organizes chromosomes into arrays of tethered chromatin loops (Kleckner 2006). As recombination proceeds, the axes of homologous chromosomes pair and are held in close physical proximity via the synaptonemal complex (Kleckner 2006). This bivalent arrangement enables meiotic recombination to complete using the homologous chromosome as a repair template, which can result in reciprocal genetic exchange, termed crossover (Villeneuve and Hillers 2001; Kleckner 2006). Formation of interhomolog crossovers creates new combinations of genetic variation along chromosomes (Villeneuve and Hillers 2001; Mercier et al. 2015), which are additionally required for balanced homolog segregation at the first meiotic division (Grelon et al. 2001). Centromere behavior is modified in meiosis, such that sister chromatid centromeres are mono-orientated to the same cell pole during the first meiotic division, whereas they are bi-orientated to opposite cell poles during the second meiotic division (Koch and Marston 2025).

The frequency of meiotic crossovers varies widely along plant chromosomes, with the presence of narrow 1 to 2 kilobase pair (kb) hotspots where recombination is elevated over the genome average (Dooner and Martínez-Férez 1997; Choi et al. 2013; Drouaud et al. 2013; Fuentes et al. 2020; Szymanska-Lejman et al. 2023). Plant crossover hotspots are influenced by both DNA sequence and chromatin, and are often found in proximity to euchromatic gene promoters and terminators (Dooner and Martínez-Férez 1997; Choi et al. 2013; Drouaud et al. 2013; Fuentes et al. 2020; Szymanska-Lejman et al. 2023). In the model plant *Arabidopsis thaliana* (hereafter Arabidopsis), the total number of crossovers can be increased by changes in the activity of *trans* regulators, for example via overexpression of the E3 ligase HEI10, or mutation of anticrossover factors (Séguéla-Arnaud et al. 2015; Ziolkowski et al. 2017). Crossovers are also subject to “assurance” and “interference,” which respectively describe that each chromosome pair receives an obligate crossover, and that double crossovers are spaced further apart than expected from uniformly random placement (Jones and Franklin 2006). HEI10 coarsening via diffusion along the synaptonemal complex provides a model to understand crossover obligation and interference (Morgan et al. 2021).

Many eukaryotic genomes also contain regions where crossovers are suppressed, including heterochromatin, mating-type loci, sex chromosomes, and the centromeres (Vincenten et al. 2015; Charlesworth 2017; Nambiar and Smith 2018; Rowan et al. 2019; Fernandes et al. 2024). Crossover suppression can be caused both by the inhibitory effects of heterochromatic marks on recombination, as well as sequence polymorphism (heterozygosity) between homologs (Charlesworth 2017; Nambiar and Smith 2018; Rowan et al. 2019; Fernandes et al. 2024). For example, acquisition of DNA methylation and H3K9me2 methylation can inhibit meiotic DSBs and crossovers within Arabidopsis recombination hotspots located close to genes (Yelina et al. 2015). The Arabidopsis centromeric and pericentromeric regions are densely DNA methylated compared to the chromosome arms, and are crossover suppressed (Rowan et al. 2019; Naish et al. 2021; Fernandes et al. 2024). However, loss of CG vs non-CG context DNA methylation in Arabidopsis has opposite effects on centromere-proximal crossover frequency. Specifically, *met1* and *ddm1* (CG) are observed to reduce proximal recombination, whereas combinations of *drm1*, *drm2*, *cmt2*, and *cmt3* (non-CG) increase crossovers (Colomé-Tatché et al. 2012; Melamed-Bessudo and Levy 2012; Mirouze et al. 2012; Yelina et al. 2015, 2012; Underwood et al. 2018; Fernandes et al. 2024). The heterochromatic histone variant H2A.W also contributes to centromere-proximal crossover suppression in Arabidopsis (Kuo et al. 2025; Son et al. 2025). Heterozygous structural variation further has a potent suppressive effect on crossovers (Rowan et al. 2019), and heterochromatic regions, including the centromeres, can be highly structurally polymorphic, even between closely related Arabidopsis accessions (Wlodzimierz et al. 2023a). Observations in budding yeast and mammals indicate that centromere-proximal crossovers can be associated with higher rates of gamete aneuploidy (Koehler et al. 1996; Lamb et al. 1996; Rockmill et al. 2006), providing a functional reason to suppress centromere-proximal crossovers. In addition, suppression of crossovers in heterochromatic regions may reduce the chance of illegitimate events, such as unequal crossover, when repetitive templates recombine (Smith 1976).

Centromeres are defined by the loading of variant nucleosomes that contain CENP-A (CENH3 in plants) instead of histone H3, which direct kinetochore formation and microtubule binding (Talbert et al. 2002; McKinley and Cheeseman 2016). In Arabidopsis, the centromeres consist of megabase arrays of 178 base pair *CEN178* satellite repeats, which are densely DNA methylated, within which a ∼1 to 2 Mb subregion is CENH3-occupied (Naish et al. 2021; Wlodzimierz et al. 2023a). The *CEN178* satellite arrays are highly structurally polymorphic between Arabidopsis accessions (Wlodzimierz et al. 2023a), reflecting the centromere paradox, where centromere sequences rapidly diversify, within and between species, despite playing a conserved and essential role during chromosome segregation (Henikoff et al. 2001). The DNA recombination and repair mechanisms that generate centromere satellite diversity are not well understood, and may include somatic and meiotic processes (Miga and Alexandrov 2021; Showman et al. 2024). Meiotic crossover maps generated from Arabidopsis Col-0×Ler-0 hybrids showed that the centromeres are contained in megabase-scale nonrecombining zones (NRZs), with proximal crossovers occurring in euchromatic gene islands embedded in the pericentromeric heterochromatin (Fernandes et al. 2024).

An evolutionary explanation for rapid centromere evolution is provided by the drive hypothesis (Fishman and Kelly 2015; Kursel and Malik 2018; Akera et al. 2019). In mammals and plants, female meiosis typically produces a single megaspore/egg that forms the zygote upon fertilization, while the remaining 3 sister gametes/spores perish (Fishman and Kelly 2015; Kursel and Malik 2018; Akera et al. 2019). Several genetic systems exist where centromere sequence variants bias their segregation into the surviving female gamete, causing segregation distortion and non-Mendelian transmission through meiosis (Fishman and Saunders 2008; Chmátal et al. 2014; Akera et al. 2017, 2019; Iwata-Otsubo et al. 2017; Kumon et al. 2021). Segregation distortion can also occur via “gene drive” in the male germ line, where linked toxin-antidote gene pairs kill gametes that do not inherit them (Nuckolls et al. 2017; Berube et al. 2024). However, the extent to which intra-species centromere polymorphism causes variation in proximal crossover frequency, and the extent to which this co-occurs with segregation distortion, is not well explored.

Arabidopsis is a powerful model for studying centromere-proximal crossover recombination and segregation distortion, as extensive natural genetic variation in satellite array structures exists, with the centromeres of multiple accessions having been fully assembled (Rabanal et al. 2022; Kang et al. 2023; Wlodzimierz et al. 2023a; Lian et al. 2024). Furthermore, fluorescent reporter systems are available that allow sensitive quantification of crossover frequency and segregation distortion across the centromeres (Berchowitz and Copenhaver 2008; Wu et al. 2015; Fernandes et al. 2024). In this study, we generated 12 Arabidopsis F_1_ hybrids by crossing a panel of diverse accessions to a common background (Col-0) that carries centromere-proximal fluorescent transgenic reporters on each chromosome, to quantify meiotic crossover frequency and segregation distortion, measuring 3,037,802 meioses in total. Compared to the Col-0 inbred parental strains, the majority of hybrids showed either significantly higher or lower centromere-proximal crossover frequency. We relate these crossover data to centromere genetic diversity and structural sequence polymorphism between the recombining chromosomes, and in a subset of 7 accessions that represent Eurasian and relict backgrounds, to epigenetic patterns of CENH3 enrichment and DNA methylation. In a minority of hybrids, we detected significant segregation distortion of centromere haplotypes through meiosis, compared to the Col-0 inbred parental strain, which occurred with or without associated changes to centromere-proximal crossover frequency. Using linear modeling, we observe that genetic variation between the centromere being measured, and the accession crossed to, and their interaction, explains ∼85% of the variation in hybrid crossover frequency, and 56% of the variation in segregation distortion. We discuss scenarios for how centromere structural polymorphism may modify recombination frequency and cause segregation distortion in Arabidopsis.

## Materials and methods

### Arabidopsis genetic stocks and growth conditions

All Col-0 FTL transgenic lines are TRAFFIC lines obtained from Prof. Scott Poethig (University of Pennsylvania, Philadelphia) (Wu et al. 2015; Poethig et al. 2022). The ANGE-B-2 and ANGE-B-10 accessions were provided by Fabrice Roux (CNRS, Toulouse) (Wlodzimierz et al. 2023a). IP-Alo-19, IP-Cat-0, and IP-Med-0 were provided by Prof. Carlos Alonso-Blanco (CNB-CSIC, Madrid) (Wlodzimierz et al. 2023a). Elk-3, Rab-1, and Tanz-1 were provided by Prof. Angela Hancock (Purdue University) (Durvasula et al. 2017), and Jm-0, Ler-0, Cvi-0, and Etna-2 were obtained from the European Arabidopsis Stock Centre. All plants were grown in controlled environment chambers at 20 °C, 60% humidity and under a 16-h photoperiod.

### Fluorescent seed scoring and calculation of crossover frequency and segregation distortion

Seeds harvested from FTL hemizygous plants were cleaned to remove plant debris, and images of a seed monolayer were captured using a Leica DFC310 FX dissecting microscope, using brightfield, or with ultraviolet filters. Images were analyzed using an adapted CellProfiler pipeline (version 4.2.5) (Stirling et al. 2021 ), which identifies seeds as primary objects and measures fluorescence intensity for each object. Thresholds for fluorescence intensity were manually set for each group of seed images, following inspection of the seed fluorescence intensity histograms. FTL crossover frequency (centiMorgan, cM) was calculated using the formula:


$$\mathrm{cM}=100\times (1\text{–}{\left[1-2(N_{G}+N_{R})/N_{T}\right]}^{1/2})$$

where *N*_G_ is the number of green-alone fluorescent seeds, *N*_R_ is the number of red-alone fluorescent seeds, and *N*_T_ is the total number of seeds counted (Ziolkowski et al. 2015). To test for significant differences in crossover frequency between the Col-0 inbred controls and each F_1_ hybrid, the *N*_R_ and *N*_G_ counts were summed across replicates for each genotype and combined with the remainder of total counts to construct 2 × 2 contingency tables for chi-square tests. To test for significant differences in segregation distortion between the Col-0 inbred controls and each F_1_ hybrid, we compared the fluorescent and nonfluorescent seed counts to inbreds, using 2 × 2 contingency tables and chi-square tests.

### Jm-0 and Elk-3 long-read DNA sequencing, genome assembly, and annotation

A single Jm-0 individual was grown at 23 °C under 16 h of light and harvested 26 d after germination. 300 mg of tissue powder was used for high-molecular weight genomic DNA extraction. The methods for DNA extraction, PacBio HiFi library preparation, genome assembly using hifiasm (Version 0.16.1-r375; Cheng et al. 2021), and quality assessment were as performed for the other 10 HiFi-based assemblies used in this study (Wlodzimierz et al. 2023a).

For Elk-3 genomic DNA extraction associated with ONT sequencing, seedlings were grown on ½ MS media and 1% sucrose. Three-week-old seedlings were moved to the dark for 48 h prior to harvesting. Approximately 2 g of tissue was used per genomic DNA extraction. Tissue was flash frozen and ground in liquid nitrogen, using a pestle and mortar for approximately 20 min. DNA was extracted using NucleoBond HMW DNA kit (Macherey-Nagel, Düren, Germany), as per the manufacturer's instructions. Library preparation followed the Nanopore SQK-LSK114 protocol and kit with the following modifications: approximately 1.2 to 1.5 µg of size-selected DNA in a volume of 48 µl was used for library preparation, the first bead clean-up after nic-repair and end-prep were replaced by Short Read Eliminator (SRE) size selection (PacBio), and libraries were sequenced in a R10.4.1 Promethion flowcell, with base-calling and DNA methylation quantification performed using dorado (v0.8.3.) with model dna_r10.4.1_e8.2_400bps_sup@v5.0.0 and –modified-bases 5mC_5hmC.

For genome assembly, reads were filtered for length and accuracy using Filtlong (Version 0.2.0) (−min_mean_q 90, –min_length 50,000), and assembled using Hifiasm (Version 0.23.0-r691), using –ont parameters (Cheng et al. 2021). Chromosomes were generated as single contigs and extracted and oriented using Ragtag (Version v2.1.0) scaffolding to the Col-CEN assembly (Naish et al. 2021; Alonge et al. 2022), and polished using medaka (version 2.0.1). For DNA methylation, reads were filtered for a minimum length of 30 kb and aggregated using Modkit pileup (Version 0.4.1), using the parameters –ignore h –mod-thresholds C:0.85.

Copies of the centromeric satellite repeat were identified using TRASH, providing a template of the *CEN178* consensus sequence (Wlodzimierz et al. 2023a, 2023b). To compare centromere structural and repeat polymorphism, we used ReDotable to generate sequence identity dot-plots, as well as SyRI (Goel et al. 2019). To map the location of syntenic insertion sites for the FTL T-DNAs in the genome assemblies of the nonreference accessions, we extracted 20 kb of sequence upstream and downstream of the insertion site from the Col-CEN assembly. These sequences were aligned to each of the accession genomes using LASTZ to identify the syntenic position in each genome. Protein sequences of the CENH3 coding region were extracted from the 13 studied *A. thaliana* accessions, and *A. lyrata* (accession MN47) (Wlodzimierz et al. 2023a), which encodes 2 CENH3 paralogues (Al1G11082 and Al1G59290) (Kawabe et al. 2006). MAFFT (https://github.com/GSLBiotech/mafft) was used to align the protein sequences, and snipit (https://github.com/aineniamh/snipit) was used for data visualization.

### Calculation of pairwise genetic diversity (*π*) in FTL regions

Genomes from each accession used in the crosses were aligned to the Col-CEN assembly (Naish et al. 2021), using Winnowmap (Version 2.03) with a k-mer size of 19 (Jain et al. 2022). Sequence variant calling was performed using BCFtools (Version 1.21) (Danecek et al. 2021). Repetitive regions in the Col-CEN assembly were masked using RepeatMasker and TRASH, including centromeric satellites, transposable elements, organelle sequences, and rDNA (Wlodzimierz et al. 2023b). Single nucleotide polymorphisms (SNPs) were filtered to exclude those overlapping repeat-masked regions, to retain strictly biallelic variants, and to limit missing data to no more than 20% across the examined accessions. Pairwise nucleotide diversity (*π*) relative to Col-CEN was calculated for each accession, within the positions of the FTL T-DNA markers on each chromosome.

### Linear models of crossover frequency and segregation distortion

As each FTL interval varies in physical size, crossover frequency was normalized by interval length based on the Col-0 genome (centiMorgans per megabases [cM/Mb]). We modeled the variation in crossover frequency using the following formula:


cM/Mb∼chromosome×accession

where “chromosome” denotes the specific FTL assayed (*CEN1-5*), and “accession” is used as the natural accession crossed to Col-0. A linear mixed effect model using “replicate” as a random variable was used to control for experimental replication. The variance explained by each term, and their interactions, was calculated using Type III ANOVA, and the sum of squares was used to estimate partial variance of each variable. Additional linear models were performed to test whether the distortion explained crossover frequency, in addition to the annotation features FTL interval size (for each accession), gene density, transposable element density, and *CEN178* satellite repeat density, which were added in a step-wise manner to the above model to determine if they would increase the proportion of variance of crossover frequency explained. Variation in segregation distortion was modeled using the same structure, where the ratio of inheritance for the red or green fluorescent transgenes was used as the response variable. The variance in distortion explained by each term was determined as above, and the same annotation features were included in models in a step-wise manner to test if they would increase the amount of variation explained.

### CENH3 ChIP-seq

Approximately 4 g of 2-week-old seedlings were ground in liquid nitrogen. Nuclei were isolated in nuclei isolation buffer (1 M sucrose, 60 mM HEPES pH 8.0, 0.6% Triton X-100, 5 mM KCl, 5 mM MgCl_2_, 5 mM EDTA, 0.4 mM PMSF, 1 mM pepstatin-A, 1×protease inhibitor cocktail) and crosslinked in 1% formaldehyde at room temperature for 25 min. The crosslinking reaction was quenched with 125 mM glycine and incubated at room temperature for a further 25 min. The nuclei were purified from cellular debris via 2 rounds of filtration through one layer of Miracloth and centrifuged at 2,500 × *g* for 25 min at 4 °C. The nuclei pellet was resuspended in EB2 buffer (0.25 M sucrose, 1% Triton X-100, 10 mM Tris–HCl pH 8.0, 10 mM MgCl_2_, 1 mM EDTA, 5 mM DTT, 0.1 mM PMSF, 1 mM pepstatin-A, 1×protease inhibitor cocktail) and centrifuged at 14,000 × *g* for 10 min at 4 °C. The pellet with the nuclei was resuspended in lysis buffer (50 mM Tris–HCl pH 8.0, 1% SDS, 10 mM EDTA, 0.1 mM PMSF, 1 mM pepstatin-A) and was sonicated using a Covaris E220 evolution with the following settings: power = 150 V, bursts per cycle = 200, duty factor = 20%, time = 60 s.

Sonicated chromatin was centrifuged at 14,000 × *g*, and the supernatant was extracted and diluted with 1×volume of ChIP dilution buffer (1.1% Triton X-100, 20 mM Tris–HCl pH 8.0, 167 mM NaCl, 1.1 mM EDTA, 1 mM pepstatin-A, 1×protease inhibitor cocktail). The chromatin was incubated overnight at 4 °C with 50 µl Protein A magnetic beads (Dynabeads, Thermo Fisher), prebound with 5 µl α-CENH3 raised to peptide acetyl-RTK HRV TRS QPR NQT DAC-amide (Eurogentec). The beads were collected on a magnetic rack and washed twice with low-salt wash buffer (150 mM NaCl, 0.1% SDS, 1% Triton X-100, 20 mM Tris–HCl pH 8.0, 2 mM EDTA, 0.4 mM PMSF, 1 mM pepstatin-A, 1×protease inhibitor cocktail) and twice with high-salt wash buffer (500 mM NaCl, 0.1% SDS, 1% Triton X-100, 20 mM Tris–HCl pH 8.0, 2 mM EDTA, 0.4 mM PMSF, 1 mM pepstatin-A, 1×protease inhibitor cocktail). Immunoprecipitated DNA–protein complexes were eluted from the beads (1% SDS, 0.1 M NaHCO_3_) at 65 °C for 15 min. Samples were reverse crosslinked by incubating with 0.24 M NaCl at 65 °C overnight. Proteins and RNA were digested with Proteinase K treatment, and RNase A, and DNA was purified by phenol:chloroform:isoamyl alcohol (25:24:1) extraction and ethanol precipitation. Library preparation followed the Tecan Ovation Ultralow System v2 library protocol. ChIP samples were PCR amplified for 12 cycles and sequenced with 150-bp paired-end reads on the Illumina platform.

### ChIP–seq data alignment, processing, and analysis

Deduplicated paired-end CENH3 ChIP–seq Illumina reads (2 × 150 bp) from Col-0, Cvi-0, Ler-0, Jm-0, Rab-1, Elk-3, and Tanz-1 were processed with Cutadapt (v.1.18) to remove adaptor sequences and low-quality bases (Phred + 33-scaled quality <20). For each accession, trimmed reads were aligned to the respective genome assembly using Bowtie2 (v.2.3.4.3), with the following settings: –very-sensitive –no-mixed –no-discordant -k 200 –maxins 1000. Read pairs with Bowtie2-assigned MAPQ of <10 were discarded using samtools (v.1.10). For retained read pairs that aligned to multiple locations, with varying alignment scores, the best alignment was selected. Alignments with more than 2 mismatches or consisting of only one read in a pair were discarded. For each dataset, bins per million mapped reads (BPM; equivalent to transcripts per million for RNA-seq data) coverage values were generated in bigWig and bedGraph formats with the bamCoverage tool from deepTools (v.3.5.0) and plotted using R. To define regions of CENH3 enrichment the start boundary was defined as the first incidence of 3 consecutive 10 kb windows which were greater than a value of log2(ChIP/Input) greater than 0.5, and the end boundary was defined by the first occurrence of 3 consecutive 10 kb windows were less than this value.

### DNA methylation profiling and analysis

DNA base-calling, alignment, and methylation calling were performed using dorado (v0.8.3.), and models dna_r10.4.1_e8.2_400bps_sup@v5.0.0 and –modified-bases 5mC_5hmC. The reads were filtered for a minimum length of 30 kb, and aggregated using Modkit pileup (Version 0.4.1), using parameters (−ignore h –mod-thresholds C:0.85). The average methylation for each DNA sequence context was calculated over 10 kb windows, using R.

## Results

### Quantifying centromere-proximal meiotic crossover frequency and segregation distortion using linked fluorescent reporter transgenes in Arabidopsis

**Fig. 1.**
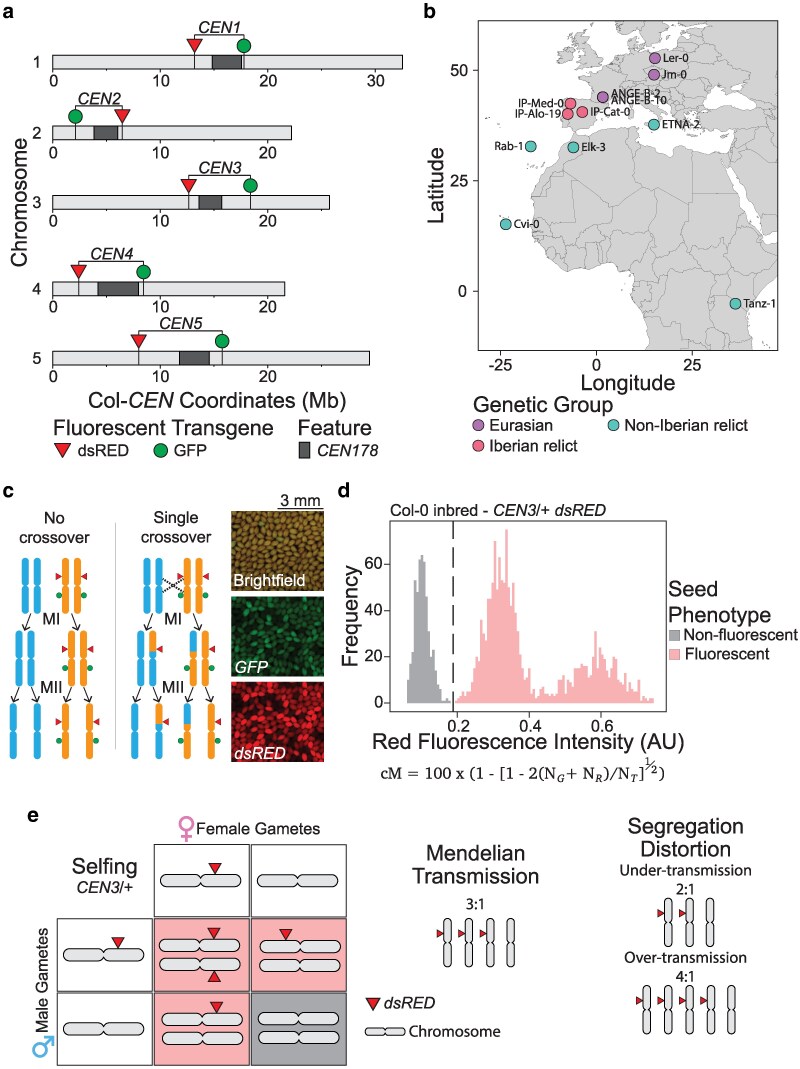
Quantifying centromere-proximal crossover frequency and segregation distortion using fluorescent reporters in Arabidopsis hybrids. a) Chromosome ideograms showing the location of FTL T-DNAs used in this study, and the *CEN178* satellite repeat arrays (dark grey), against the Col-CEN genome assembly (Naish et al. 2021). Red and green triangles indicate the location of FTL T-DNAs expressing dsRED and GFP, respectively (Wu et al. 2015; Poethig et al. 2022). b) Geographic map showing the origin of the Arabidopsis accessions studied. Accession locations are color-coded by SNP PCA chromosome arm similarity groups (Wlodzimierz et al. 2023a): Eurasian (purple), Iberian relict (pink), and non-Iberian relict (turquoise). The geographic map was obtained from R package ggmap. c) Diagram showing genetic segregation of a hemizygous FTL chromosome through meiosis I (MI) and II (MII). Homologous chromosomes are shown in blue and orange, with the orange genotype representing Col-0 FTL chromosomes, and FTL transgenes shown by red and green triangles. Two segregation scenarios are shown, either with or without a single crossover within the FTL interval. Alongside are representative micrographs of *FTL/+* hemizygous seed imaged in brightfield or visualizing red or green fluorescence. Scale bar = 3 mm. d) A representative histogram of red fluorescence (dsRED) of seed from a self-fertilized *CEN3/+* hemizygous plant. The dotted line indicates the threshold that divides nonfluorescent (light grey) and red fluorescent (red) seed. To calculate crossover frequency (cM) within the FTL genetic interval, the formula shown is applied to the counts of green (*N*_G_) and red (*N*_R_) fluorescent seed and the total seed (*N*_T_). e) Punnett square showing the expected male and female gametes produced by an FTL hemizygote, and the expected Mendelian inheritance ratio of 3 fluorescent to 1 nonfluorescent progeny, following self-fertilization. On the right, a diagram indicating that higher or lower ratios of fluorescent to nonfluorescent seed can indicate segregation distortion in progeny seed.

In Arabidopsis, segregation of linked, hemizygous Fluorescent-Tagged Line (FTL) T-DNAs that express different colors of fluorescent protein in the pollen, or nonmaternal seed tissues, can be used to quantify meiotic crossover frequency between the transgene insertions (Melamed-Bessudo et al. 2005; Wu et al. 2015; Poethig et al. 2022). Based on the locations of the centromeric *CEN178* satellite arrays in the Col-CEN genome assembly (Naish et al. 2021), we selected pairs of FTL T-DNAs generated in the Col-0 accession that define physical intervals that span each of the 5 centromeres (Fig. 1a, Supplementary Fig. 1 and Supplementary Table 1) (Wu et al. 2015; Poethig et al. 2022). In the Col-CEN assembly, the FTL intervals range from 4.36 to 7.76 megabases (Mb) (Fig. 1a, Supplementary Fig. 1 and Supplementary Table 1). In addition to the *CEN178* satellite arrays, the FTL *CEN* intervals contain varying amounts of pericentromeric heterochromatin, *5S* rDNA copies, genes, and transposable elements (Fig. 1a, Supplementary Fig. 1 and Supplementary Table 1) (Naish et al. 2021). When FTL T-DNAs are linked and hemizygous, and the plant is self-fertilized, the number of seeds with each fluorescent phenotype (red and green: red-alone: green-alone: no color) can be used to quantify meiotic crossover frequency (Fig. 1, c and d) (Melamed-Bessudo et al. 2005; Francis et al. 2007; Wu et al. 2015; Poethig et al. 2022). If FTL hemizygous plants are self-fertilized, both male and female meiotic crossovers are assayed (Melamed-Bessudo et al. 2005; Francis et al. 2007; Wu et al. 2015; Poethig et al. 2022). In Col-0 inbreds, the centromere-spanning FTL intervals have recombination rates in the range of 0.86 to 2.00 cM/Mb (Supplementary Table 1), which is lower than the chromosome arm average of 3.80 cM/Mb measured from Col-0×Ler-0 hybrids (Fernandes et al. 2024), consistent with centromere-proximal suppression of crossover frequency.

FTL T-DNAs can also be used to quantify segregation distortion between generations. When an FTL hemizygote undergoes meiosis, the Mendelian expectation is that exactly half of the gametes will carry each T-DNA (Fig. 1, c and e). As our F_2_ seed samples represent self-fertilization of male and female meiotic products, the Mendelian expectation is that two thirds of the seeds should show fluorescence for either T-DNA; i.e. two thirds of the seeds should be red-fluorescent, and two thirds should be green-fluorescent (Fig. 1e). In the case of segregation distortion, we would expect to observe either an excess of seeds with red and green fluorescence if the Col-0 centromere interval is over-transmitting, or fewer colored seeds if it is under-transmitting (Fig. 1e). As the centromere is in linkage with the markers, we would expect correlated inheritance behavior by both red and green FTL markers when centromere-linked distortion occurs. In this way, we sought to use the FTL intervals to simultaneously quantify meiotic crossover frequency, and segregation distortion, in centromere-proximal intervals of a diverse panel of Arabidopsis F_1_ hybrids, compared to Col-0 inbred lines.

To investigate the effect of natural variation on FTL centromere-proximal crossover frequency, we selected a diverse panel of 12 natural Arabidopsis accessions for analysis (Fig. 1b and Supplementary Tables 1 and 2). These accessions capture diversity from Eurasian (ANGE-B-2, ANGE-B-10, Ler-0, Col-0, and Jm-0), Iberian relict (IP-Cat-0, IP-Med-0, and IP-Alo-19), and non-Iberian relict (Etna-2, Tanz-1, Elk-3, Rab-1, and Cvi-0) genome-wide similarity groups (Fig. 1b and Supplementary Table 2) (Naish et al. 2021; Wlodzimierz et al. 2023a). The Col-0 reference strain carrying the fluorescent reporters is a member of the Eurasian genetic group. Centromere-complete genome assemblies have been published for these accessions (Wlodzimierz et al. 2023a), except for Jm-0 and Elk-3. Therefore, Jm-0 was HiFi sequenced to 111× coverage, and Elk-3 was Oxford Nanopore (ONT) sequenced to 1,151× coverage with a subset of >50 kb reads used for genome assembly (248× coverage) using hifiasm (Cheng et al. 2021). For Elk-3, all chromosomes were assembled as single contigs, while for Jm-0 chromosomes there were 9 gaps in total. For each genome assembly, we used the TRASH algorithm to identify the position and size of the centromeric *CEN178* arrays, as well as annotating genes using Helixer, and transposons using EDTA (Supplementary Table 1) (Ou et al. 2019; Stiehler et al. 2021; Wlodzimierz et al. 2023b).

In our experiments, homozygous FTL lines in the Col-0 accession were crossed with the 12 inbred accessions to generate replicate F_1_ hybrids. The F_1_ hybrids were allowed to self-fertilize and their seeds analyzed using fluorescence microscopy to quantify both crossover frequency, and segregation distortion (Fig. 1, c to e and Supplementary Fig. 2). For each genotype, we scored an average of 12 F_1_ replicates, with on average 2,005 seed analyzed per replicate, and 1,518,901 seed analyzed in total, representing 3,037,802 meioses (Supplementary Tables 3 to 7). We first consider each centromere individually, and then together, and relate their genetic and epigenetic organization to variation in centromere-proximal meiotic crossover frequency and segregation distortion.

### Meiotic crossover frequency and segregation distortion within *CENTROMERE1*

**Fig. 2.**
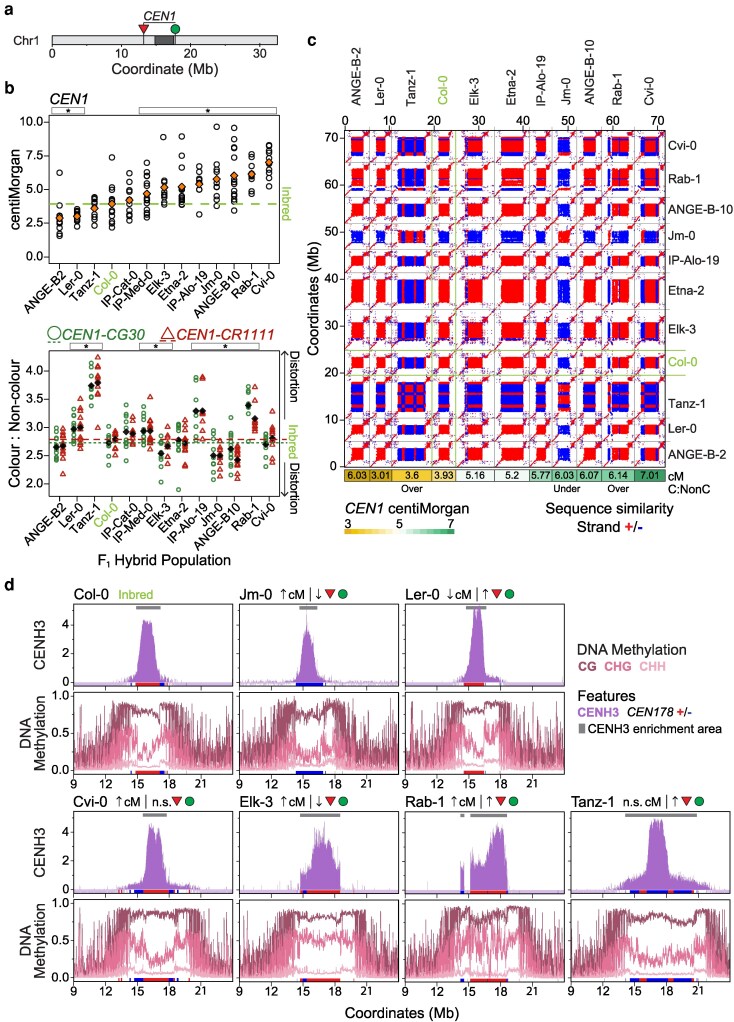
Meiotic crossover frequency, segregation distortion, and genetic and epigenetic structure within *CENTROMERE1.* a) Chromosome ideogram showing the location of FTL T-DNAs used in this study, together with the position of the *CEN178* satellite repeat arrays (dark grey), shown against the Col-CEN genome assembly (Naish et al. 2021). Red and green triangles indicate the location of FTL T-DNAs expressing dsRED and GFP in seed from the *napA* promoter, respectively (Wu et al. 2015; Poethig et al. 2022). b) Crossover frequency (cM) values of individual replicates of F_1_ hybrids between the accession listed on the *x*-axis, and the *CEN1* FTL reporters in a Col-0 background. Mean values are shown by orange diamonds. The inbred Col-0×Col-0 FTL control is highlighted in green, via the horizontal dashed line. Stars at the top of the plot indicate hybrids with significantly different crossover frequencies to the inbred strain based on chi-square tests (*P* < 0.05). Beneath, the same data are analyzed for the ratios of green (circles, short dashed line) and red fluorescent (triangles, long dashed line) seed to nonfluorescent seed and presented in the same way. c) Sequence similarity dot plots comparing the *CEN178* arrays from a subset of the accessions analyzed in b. Red and blue shading indicate sequence similarity on forward and reverse strands, using a 161 base pair window. Beneath the dotplot, the cM value for the corresponding *CEN1* hybrid from b is shown, which is proportionally shaded. Additionally, beneath the dot plot are text indicators showing which accessions showed segregation distortion in b (Color:Noncolor, C:NonC). d) For the Col-0, Jm-0, and Ler-0 Eurasian accessions, and the Cvi-0, Elk-3, Rab-1, and Tanz-1 relict accessions, the CENH3 ChIP-seq enrichment (upper, purple) and DNA methylation (lower) in CG (dark pink), CHG (pink), and CHH (light pink) contexts across the *CEN1* region are shown. The position of *CEN178* arrays is indicated by ticks on the x-axis on the forward (red) or reverse (blue) strand.

We used the *CTL1.48* interval traffic line in the Col-0 background (Wu et al. 2015; Poethig et al. 2022), which contains a red-fluorescent protein encoding T-DNA *CR1111* at base pair position 13,216,744, and a green-fluorescent protein encoding T-DNA *CG30* at position 17,784,719 on chromosome 1 of the Col-CEN genome assembly (Fig. 2a, Supplementary Fig. 1 and Supplementary Table 1). For the purposes of this study, we renamed *CTL1.48* as *CENTROMERE1* (*CEN1*), which is 4.57 Mb long in the Col-CEN assembly. Relative to previous Col-0×Ler-0 genetic maps, 79% (3.60 Mb) of the *CEN1* interval corresponds to NRZ, with the remainder (0.96 Mb) corresponding to low-recombining zone (LRZ) (Fernandes et al. 2024). For each genotype, we quantified *CEN1* crossover frequency (cM) from replicate individuals, using the counts of red alone (*N*_R_) and green alone (*N*_G_) seeds, compared to the total number (Supplementary Table 3) (Melamed-Bessudo et al. 2005; Francis et al. 2007; Wu et al. 2015; Poethig et al. 2022). To test for significant differences in crossover frequency between the Col-0 inbred controls and each F_1_ hybrid, the *N*_R_ and *N*_G_ counts were summed across replicates for each genotype and combined with total counts to construct 2 × 2 contingency tables, and chi-square tests were performed.

Compared to Col-0 inbreds, we observed that ANGE-B-2×Col-0 and Ler-0×Col-0 F_1_ hybrids had significantly lower crossover frequency, while recombination was significantly higher in 8 hybrids (chi-square tests, all *P* < 2.05 × 10^−5^) (Fig. 2b and Supplementary Table 3). Interestingly, the 2 highest-recombining hybrids, Rab-1×Col-0 and Cvi-0×Col-0, are between diverged relict accessions and the Eurasian Col-0 parent, with larger *CEN178* centromere arrays that contain 8,523 and 7,717 additional copies, respectively (Fig. 2, b and c and Supplementary Table 1). In contrast, the intra-Eurasian low-recombining ANGE-B-2×Col-0 and Ler-0×Col-0 hybrids are from accessions with *CEN178* arrays that are more similar in size and structure to Col-0 (Fig. 2, b and c and Supplementary Table 1). However, overall there was no significant correlation between *CEN1* crossover frequency and the size difference of the hybrid *CEN178* arrays (defined as the difference in *CEN178* copies between the centromeres) (*r* = 0.023, *P* = 0.120) (Fig. 2, b and c, Supplementary Fig. 3 and Supplementary Table 1). For example, the Tanz-1 accession, which has the largest *CEN1 CEN178* arrays (34,131 copies), which are arranged in a series of inverted blocks, produced F_1_ hybrids with a crossover frequency not significantly different to Col-0 inbreds (Fig. 2, b and c, Supplementary Fig. 4 and Supplementary Table 1). Jm-0 was also notable as it bears a *CEN178* array on the opposite strand to Col-0 and yet produced hybrids with a significantly higher crossover frequency (*P* = 1.44 × 10^−18^) (Fig. 2, b and c, Supplementary Fig. 4 and Supplementary Table 1). These observations indicate that the size and structure of the *CEN178* arrays do not simply predict recombination frequency outcome.

In addition to assessing how satellite array polymorphism correlates with centromere-proximal recombination, we calculated pairwise nucleotide diversity (*π*) within the FTL regions, using high-confidence biallelic SNPs, as an additional measure of the genetic diversity between the hybrid accessions and Col-0. As for *CEN178* copy number polymorphism, we did not observe a significant correlation between FTL interval *π* and crossover frequency (Supplementary Fig. 3 and Supplementary Table 1). We conclude that although substantial variation in *CEN1* centromere-proximal crossover frequency exists between Arabidopsis hybrids, this is not significantly correlated with FTL nucleotide diversity, or structural variation in the *CEN178* arrays.

We next sought to use the FTL seed data to investigate segregation distortion. We calculated the ratio of red (*N*_R_) to nonred fluorescent (*N*_NR_), and green (*N*_G_) to nongreen fluorescent (*N*_NG_) seeds. For each F_1_ hybrid sample we compared the fluorescent and nonfluorescent seed counts to those from Col-0 inbreds, using 2 × 2 contingency tables, and performed chi-square tests to assess significant differences (Fig. 2b). When both red and green inheritance ratios were significantly different from Col-0 inbreds, with the same direction of change, we considered this as evidence of centromere-linked segregation distortion. We observed that 5 F_1_ hybrids, derived from the Ler-0, Tanz-1, IP-Med-0, IP-Alo-19, and Rab-1 accessions, showed significant over-transmission of the Col-0 centromere *CEN1* FTL interval (chi-square tests, all *P* ≤ 0.011), whereas, 3, with Elk-3, Jm-0, and ANGE-B-10, showed significant under-transmission (chi-square tests, all *P* ≤ 0.026), compared to inbreds (Fig. 2b and Supplementary Table 1). The direction and strength of distorted inheritance did not correlate with changes to meiotic crossover frequency (Supplementary Fig. 2). For example, the strongest distortion occurred against Tanz-1, where there was no significant change to *CEN1* crossover frequency, as well as in the highly recombining IP-Alo-19×Col-0 and Rab-1×Col-0 hybrids (Fig. 2b and Supplementary Table 1). Equally, there was no correlation between the strength or direction of distorted inheritance and how different the hybrid *CEN178* arrays were in size, or with FTL nucleotide diversity (*π*) (Fig. 2, b and c, Supplementary Figs. 3 and 4 and Supplementary Table 1). Notably, the Tanz-1 centromere, which has the largest *CEN178* arrays, showed the greatest under-transmission against Col-0 (Fig. 2, b and c, Supplementary Figs. 3 and 4 and Supplementary Table 1). Together, this reveals distorted *CEN1* inheritance in Arabidopsis hybrids, compared to inbreds, but which was not simply explained by *CEN178* structural variation, FTL nucleotide diversity, or centromere-proximal crossover frequency.

We next examined epigenetic organization of the centromeres. In a subset of 7 parental accessions, the Eurasians Col-0, Ler-0, and Jm-0, and the relicts Cvi-0, Tanz-1, Elk-3, and Rab-1, we compared ChIP-seq enrichment of the centromeric histone CENH3, together with DNA methylation in CG, CHG, and CHH sequence contexts across *CEN1* (Wlodzimierz et al. 2023a). We defined zones of CENH3 enrichment, and in all cases observed focused localization within the *CEN178* satellite arrays (Fig. 2d and Supplementary Table 1). We found CENH3 occupancy to be enriched in regions that were between 1.67 and 6.74 Mb wide, and typically with a single peak (Fig. 2d). Exceptions included the relict accessions Tanz-1 and Rab-1; in Tanz-1, CENH3 was enriched in a relatively broad 6.74 Mb region, although with strongest enrichment within a single *CEN178* inversion block, whereas in Rab-1, CENH3 was loaded asymmetrically within the largest satellite array (Fig. 2d). In each accession, the centromeres are embedded in highly DNA methylated pericentromeric regions (Fig. 2d). The *CEN178* arrays themselves show similar CG context DNA methylation relative to the peri-centromeres, but with reduced CHG and CHH methylation (Fig. 2d). Taken together, this indicates stability of epigenetic organization in the Arabidopsis centromeres, with respect to CENH3 loading, despite a high level of structural genetic variation in the underlying sequences. Overall, the size of the CENH3 zone of enrichment, and the pattern of DNA methylation, did not explain changes to crossover frequency, or the strength or direction of segregation distortion (Supplementary Fig. 5 and Supplementary Table 1).

### Meiotic crossover frequency and segregation distortion within *CENTROMERE2*

**Fig. 3.**
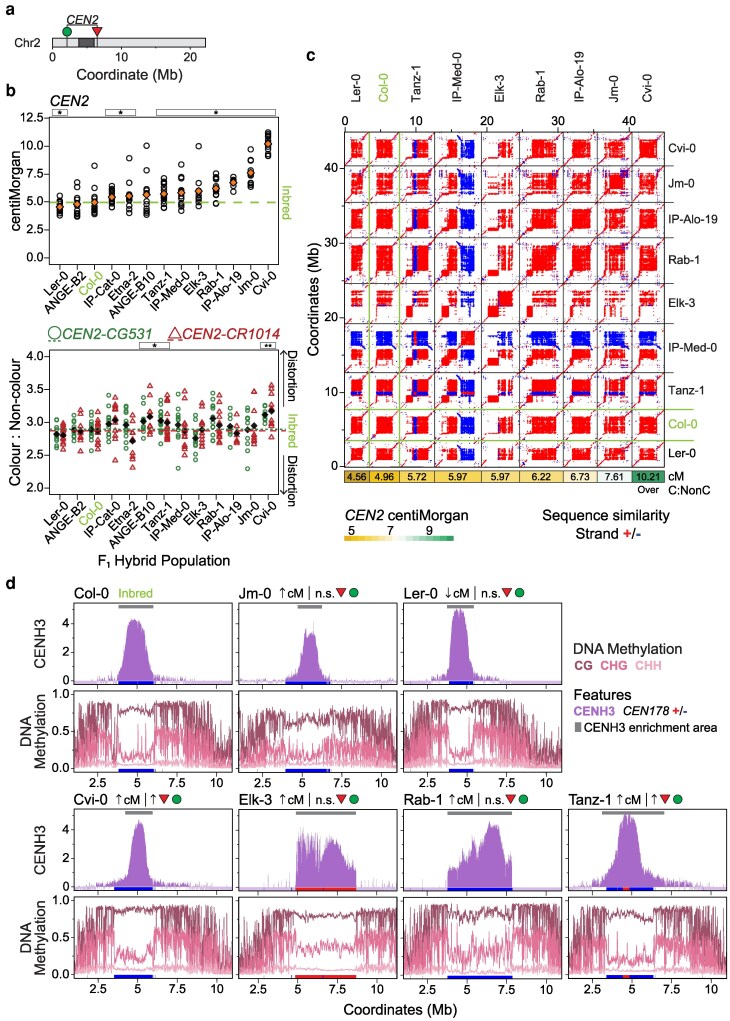
Meiotic crossover frequency, segregation distortion, and genetic and epigenetic structure within *CENTROMERE2.* a) Chromosome ideogram showing the location of FTL T-DNAs used in this study, together with the position of the *CEN178* satellite repeat arrays (dark grey), against the Col-CEN genome assembly (Naish et al. 2021). Red and green triangles indicate the location of FTL T-DNAs expressing dsRED and GFP in seed from the *napA* promoter, respectively (Wu et al. 2015; Poethig et al. 2022). b) Crossover frequency (cM) values of individual replicates of F_1_ hybrids between the accession listed on the *x*-axis, and the *CEN2* FTL reporters in a Col-0 background. Mean values are shown by orange diamonds. The inbred Col-0×Col-0-FTL control is highlighted in green, via the horizontal dashed line. Stars at the top of the plot indicate hybrids with significantly different crossover frequencies to the inbred strain based on chi-square tests (*P* < 0.05). Beneath, the same data are analyzed for the ratios of green (circles, short dashed line) and red (triangles, long dashed line) fluorescent seed to nonfluorescent seed and presented in the same way. c) Sequence similarity dot plots comparing the *CEN178* arrays from a subset of the accessions analyzed in b. Red and blue shading indicate sequence similarity on forward and reverse strands, using a 161 base pair window. Beneath the dotplot, the cM value for the corresponding *CEN2* hybrid from b is shown, which is proportionally shaded. Additionally, beneath the dot plot are text indicators showing which accessions showed segregation distortion in b (Color:Noncolor, C:NonC). d) For the Col-0, Jm-0, and Ler-0 Eurasian accessions, and the Cvi-0, Elk-3, Rab-1, and Tanz-1 relict accessions, the CENH3 ChIP-seq enrichment (upper, purple) and DNA methylation (lower) in CG (dark pink), CHG (pink), and CHH (light pink) contexts across the *CEN1* region are shown. The position of *CEN178* arrays is indicated by ticks on the *x*-axis on the forward (red) or reverse (blue) strand.

For chromosome 2, we used the *CTL2.16* traffic line (Wu et al. 2015; Poethig et al. 2022), which consists of a red fluorescent protein encoding T-DNA *CR1014* located at base pair position 6,472,440 of chromosome 2 in the Col-CEN genome assembly, and a green-fluorescent protein encoding T-DNA *CG531* at position 2,108,979 bp. For the purposes of this study, we renamed *CTL2.16* as *CENTROMERE2* (*CEN2*), which is 4.36 Mb long in the Col-CEN assembly (Fig. 3a, Supplementary Fig. 1 and Supplementary Table 1). Relative to previous Col-0×Ler-0 genetic maps, 76% (3.30 Mb) of the *CEN2* interval corresponds to NRZ, with the remainder corresponding to LRZ (17.2%, 0.75 Mb) and chromosome arm (7.1%, 0.31 Mb) sequences (Fernandes et al. 2024). In *CEN2*, we observed a single hybrid (with Ler-0) that had significantly lower crossover frequency than Col-0 inbreds, whereas 9 hybrids were significantly higher (chi-square tests, all *P* ≤ 0.023) (Fig. 3b, Supplementary Tables 1 and 4). As for *CEN1*, we did not observe a significant pairwise correlation between *CEN178* array size, structural polymorphism, or FTL interval nucleotide diversity, and *CEN2* crossover frequency in the hybrids (Fig. 3, b and c, Supplementary Figs. 3 and 6 and Supplementary Table 1). In hybrids with the divergent relict Tanz-1 and IP-Med-0 accessions, which have structurally polymorphic *CEN178* arrays relative to Col-0, including inversions, we observed significantly elevated hybrid crossovers, compared to Col-0 inbreds (chi-square tests, both *P* ≤ 3.45 × 10^−4^) (Fig. 3, b and c, Supplementary Fig. 6 and Supplementary Table 1), indicating again that satellite array genetic divergence is not a barrier to elevated centromere-proximal crossover frequency in Arabidopsis hybrids.

Compared to *CEN1*, fewer of the *CEN2* hybrids showed significant segregation distortion, with ANGE-B-10×Col-0, Tanz-1×Col-0, and Cvi-0×Col-0 hybrids showing over-transmission of the Col-0 centromere (chi-square tests, all *P* ≤ 0.034) (Fig. 3b and Supplementary Table 1). Similar to *CEN1*, the incidence of segregation distortion did not correlate either with FTL nucleotide diversity, *CEN178* array size differences, or centromere-proximal crossover frequency (Fig. 3, b and c, Supplementary Figs. 3 and 6 and Supplementary Table 1).

We compared the epigenetic landscape across *CEN2* and observed 4 accessions with zones of CENH3 enrichment 1.53 to 2.19 Mb, whereas the relicts Tanz-1, Rab-1, and Elk-3 showed larger *CEN178* arrays that contained broader 3.42 to 4.06 Mb CENH3-occupied regions (Fig. 3d and Supplementary Table 1). The Rab-1 and Elk-3 hybrids both show significantly higher *CEN3* crossover frequency, but no evidence of distortion (Fig. 3b). In each of the 7 accessions, the region of CENH3 enrichment within *CEN2* coincides with high CG DNA methylation, and depleted CHG and CHH methylation in the *CEN178* arrays relative to the peri-centromeres (Fig. 3d). This reinforces the conclusion that although significant variation in centromere-proximal crossover frequency and segregation distortion exists between Arabidopsis hybrids, this is not easily explained by satellite array polymorphism, nucleotide diversity, or the zones of CENH3 enrichment and DNA methylation states.

### Meiotic crossover frequency and segregation distortion within *CENTROMERE3*

**Fig. 4.**
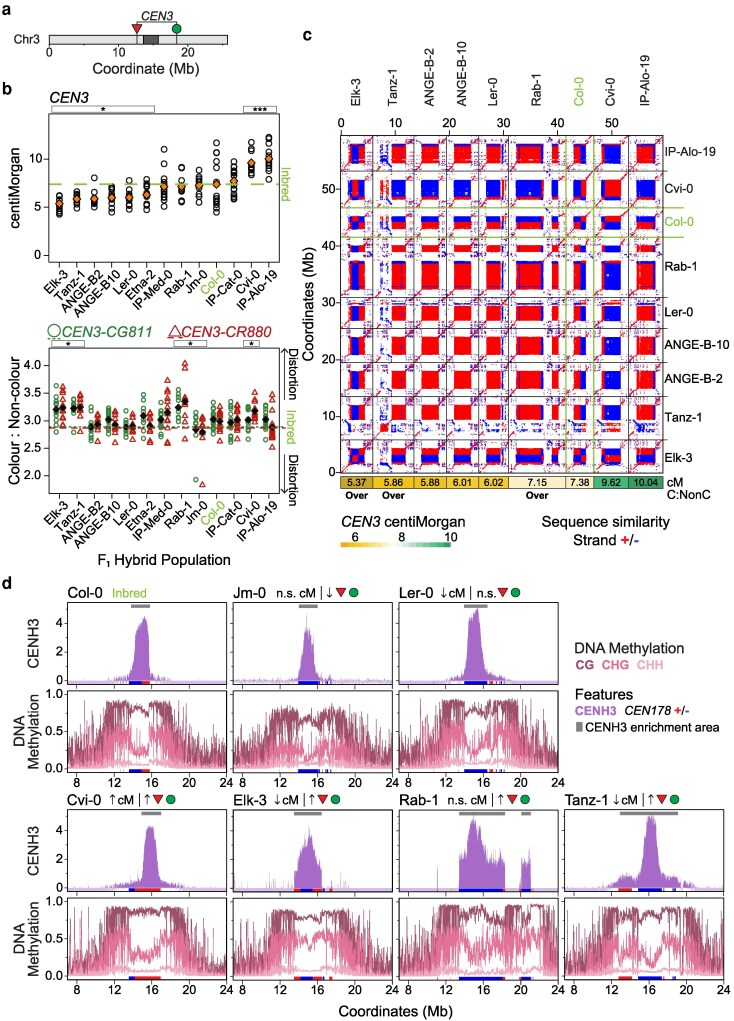
Meiotic crossover frequency, segregation distortion, and genetic and epigenetic structure within *CENTROMERE3.* a) Chromosome ideogram showing the location of FTL T-DNAs used in this study, together with the position of the *CEN178* satellite repeat arrays (dark grey), against the Col-CEN genome assembly (Naish et al. 2021). Red and green triangles indicate the location of FTL T-DNAs expressing dsRED and GFP in seed from the *napA* promoter, respectively (Wu et al. 2015; Poethig et al. 2022). b) Crossover frequency (cM) values of individual replicates of F_1_ hybrids between the accession listed on the *x*-axis, and the *CEN3* FTL reporters in a Col-0 background. Mean values are shown by orange diamonds. The inbred Col-0×Col-0-FTL control is highlighted in green, via the horizontal dashed line. Stars at the top of the plot indicate hybrids with significantly different crossover frequencies to the inbred strain based on chi-square tests (*P* < 0.05). Beneath, the same data are analyzed for the ratios of green (circles, short dashed line) and red (triangles, long dashed line) fluorescent seed to nonfluorescent seed and presented in the same way. c) Sequence similarity dot plots comparing the *CEN178* arrays from a subset of the accessions analyzed in b. Red and blue shading indicate sequence similarity on forward and reverse strands, using a 161 base pair window. Beneath the dotplot, the cM value for the corresponding *CEN3* hybrid from b is shown, which is proportionally shaded. Additionally, beneath the dot plot are text indicators showing which accessions showed segregation distortion in b (Color:Noncolor, C:Non-C). d) For the Col-0, Jm-0, and Ler-0 Eurasian accessions, and the Cvi-0, Elk-3, Rab-1, and Tanz-1 relict accessions, the CENH3 ChIP-seq enrichment (upper, purple) and DNA methylation (lower) in CG (dark pink), CHG (pink), and CHH (light pink) contexts across the *CEN1* region are shown. The position of *CEN178* arrays is indicated by ticks on the *x*-axis on the forward (red) or reverse (blue) strand.

To study chromosome 3, we combined the red-encoding FTL T-DNA *CR880*, located at base pair position 12,652,136 of chromosome 3 in the Col-CEN genome assembly, with a green-encoding T-DNA *CG811* at position 18,395,802, and named this interval *CENTROMERE3* (*CEN3*), which is 5.743 Mb long in Col-CEN (Fig. 4a, Supplementary Fig. 1 and Supplementary Table 1) (Wu et al. 2015; Poethig et al. 2022). Relative to previous Col-0×Ler-0 genetic maps, 52% (3.0 Mb) of the *CEN3* interval corresponds to NRZ, with the remainder corresponding to LRZ (34.2%, 1.1 Mb) and chromosome arm (13.6%, 0.78 Mb) sequences (Fernandes et al. 2024). In contrast to *CEN1* and *CEN2*, where the majority of hybrids showed significantly higher crossover frequency compared to inbreds, in *CEN3*, 6 hybrids were lower, and 2 were higher (chi-square tests, all *P* ≤ 2.73 × 10^−5^) (Fig. 4b and Supplementary Table 5). Interestingly, hybrids with the Cvi-0 and IP-Alo-19 relict accessions again showed elevated crossover frequency, despite structural polymorphism in the *CEN178* arrays, compared to Col-0 inbreds (Fig. 4, b and c, Supplementary Figs. 3 and 7 and Supplementary Table 1). In Col-0, the *CEN3 CEN178* arrays have an inverted array structure (Naish et al. 2021), with inversions also present in the Elk-3, Ler-0, Rab-1, Cvi-0, and IP-Alo-19 satellite arrays, which together include the parents of both the highest (IP-Alo-19) and lowest (Elk-3) recombining hybrids, arguing that the *CEN178* inversion structure does not control crossover frequency (Fig. 4, b and c, Supplementary Fig. 7 and Supplementary Table 1). Three hybrids with relict parents (Elk-3, Tanz-1, and Rab-1) showed significant over-transmission of the Col-0 centromere as monitored by FTL inheritance ratios (chi-square test, all *P* ≤ 0.0031), whereas the Eurasian Jm-0×Col-0 hybrid showed significant Col-0 under-transmission (chi-square tests, both *P* ≤ 0.0011) (Fig. 4b and Supplementary Table 1). In the Rab-1×Col-0 hybrids, the *CEN3 CEN178* array is substantially larger than in Col-0 (2.14 vs 5.9 Mb), indicating again that larger array size does not associate with stronger distorted inheritance in Arabidopsis (Fig. 4c and Supplementary Table 1). At the chromatin level, Col-0 shows a single 2.01 Mb region of CENH3 enrichment, whereas Rab-1 shows CENH3 enrichment on discrete large (4.9 Mb) and small (0.99 Mb) *CEN178* arrays (Fig. 4d and Supplementary Table 1), which have the potential to contribute to distorted inheritance. With respect to CENH3 loading, Cvi-0, Ler-0, and Tanz-1 ChIP-seq enrichment was observed within *CEN178* subarrays that were mainly arranged on a single strand, whereas in Col-0 and Elk-3, CENH3 loading spans inverted satellite arrays (Fig. 4d).

### Meiotic crossover frequency and segregation distortion within *CENTROMERE4*

**Fig. 5.**
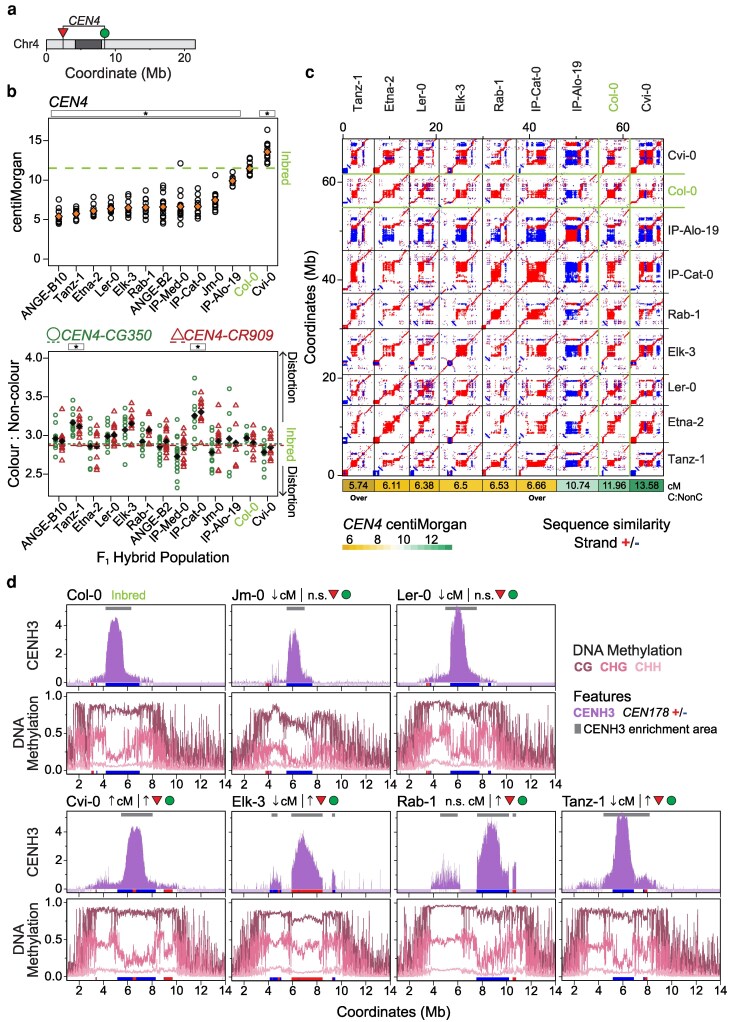
Meiotic crossover frequency, segregation distortion, and genetic and epigenetic structure within *CENTROMERE4.* a) Chromosome ideogram showing the location of FTL T-DNAs used in this study, together with the position of the *CEN178* satellite repeat arrays (dark grey), against the Col-CEN genome assembly (Naish et al. 2021). Red and green triangles indicate the location of FTL T-DNAs expressing dsRED and GFP in seed from the *napA* promoter, respectively (Wu et al. 2015; Poethig et al. 2022). b) Crossover frequency (cM) values of individual replicates of F_1_ hybrids between the accession listed on the *x*-axis, and the *CEN4* FTL reporters in a Col-0 background. Mean values are shown by orange diamonds. The inbred Col-0×Col-0-FTL control is highlighted in green, via the horizontal dashed line. Stars at the top of the plot indicate hybrids with significantly different crossover frequencies to the inbred strain based on chi-square tests (*P* < 0.05). Beneath, the same data are analyzed for the ratios of green (circles, short dashed line) and red (triangles, long dashed line) fluorescent seed to nonfluorescent seed and presented in the same way. c) Sequence similarity dot plots comparing the *CEN178* arrays from a subset of the accessions analyzed in b. Red and blue shading indicate sequence similarity on forward and reverse strands, using a 161 base pair window. Beneath the dotplot, the cM value for the corresponding *CEN4* hybrid from b is shown, which is proportionally shaded. Additionally, beneath the dot plot are text indicators showing which accessions showed segregation distortion in b (Color:Non-color, C:Non-C). d) For the Col-0, Jm-0, and Ler-0 Eurasian accessions, and the Cvi-0, Elk-3, Rab-1, and Tanz-1 relict accessions, the CENH3 ChIP-seq enrichment (upper, purple) and DNA methylation (lower) in CG (dark pink), CHG (pink), and CHH (light pink) contexts across the *CEN1* region are shown. The position of *CEN178* arrays is indicated by ticks on the *x*-axis on the forward (red) or reverse (blue) strand.

For chromosome 4, we used the *CTL4.12* interval traffic line (Wu et al. 2015; Poethig et al. 2022), which consists of a red-encoding T-DNA *CR909* at base pair position 2,437,418 of chromosome 4 in the Col-CEN genome assembly, and a green-encoding T-DNA *CG350* at position 8,480,638. For the purposes of this study, we renamed *CTL4.12* as *CENTROMERE4* (*CEN4*), which is 6.04 Mb long in Col-CEN (Fig. 5a, Supplementary Fig. 1 and Supplementary Table 1). Relative to previous Col-0×Ler-0 genetic maps, 67.8% (4.1 Mb) of the *CEN4* interval corresponds to NRZ, with the remainder corresponding to LRZ (26.4%, 1.59 Mb), and chromosome arm (5.8%, 0.35 Mb) sequences (Fernandes et al. 2024). For *CEN4*, the majority of hybrids (11 of 12) showed significantly reduced crossover frequency compared to inbreds, with the only exception being the relict Cvi-0×Col-0 hybrid, which had significantly higher recombination (chi-square test, *P* = 1.31 × 10^−4^) (Fig. 5b and Supplementary Table 6). Col-0 is documented to have a large “knob” inversion on the short arm of chromosome 4 that spans the *CR909* FTL T-DNA (Fransz et al. 2000, 2016), which is not shared with any of the 12 parental accessions crossed to (Supplementary Fig. 8). This inversion almost certainly contributes substantially to the widespread suppressed crossover frequency in the *CEN4* hybrids tested here.

Two relict accessions, Tanz-1 and IP-Cat-0, produced hybrids with significant over-transmission of the Col-0 FTL centromere (chi-square tests, both *P* ≤ 1.80 × 10^−3^) (Fig. 5b and Supplementary Table 1). Both accessions share the knob inversion but are otherwise different from one another in *CEN178* array size and structure (Fig. 5b, Supplementary Fig. 8 and Supplementary Table 1). Amongst the 7 accessions profiled at the level of chromatin, the relicts Rab-1 and Tanz-1 showed the presence of multiple distinct *CEN178* arrays, one of which contained the majority of CENH3 in each case (Fig. 5d). In both Rab-1×Col-0 and Tanz-1×Col-0 hybrids, the smaller Col-0 *CEN178* array showed biased segregation distortion in its own favor (Fig. 5b and Supplementary Table 1), further reinforcing that larger satellite arrays in Arabidopsis do not associate with distorted inheritance in their own favor.

### Meiotic crossover frequency and segregation distortion within *CENTROMERE5*

**Fig. 6.**
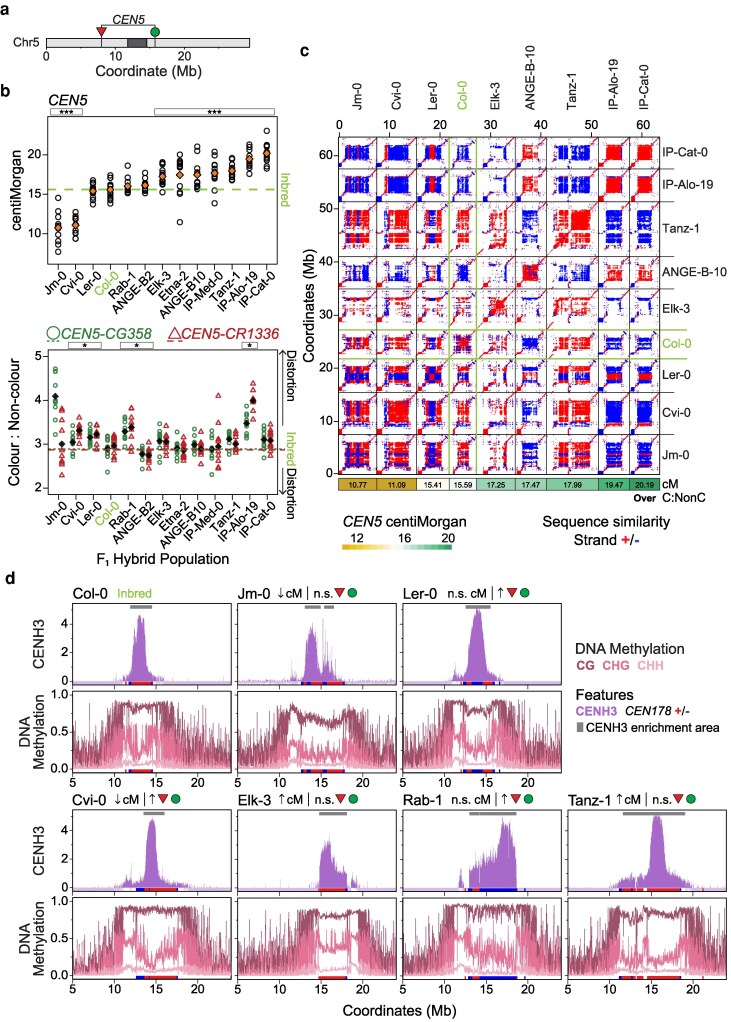
Meiotic crossover frequency, segregation distortion, and genetic and epigenetic structure within *CENTROMERE5.* a) Chromosome ideogram showing the location of FTL T-DNAs used in this study, together with the position of the *CEN178* satellite repeat arrays (dark grey), against the Col-CEN genome assembly (Naish et al. 2021). Red and green triangles indicate the location of FTL T-DNAs expressing dsRED and GFP in seed from the *napA* promoter, respectively (Wu et al. 2015; Poethig et al. 2022). b) Crossover frequency (cM) values of individual replicates of F_1_ hybrids between the accession listed on the *x*-axis, and the *CEN5* FTL reporters in a Col-0 background. Mean values are shown by orange diamonds. The inbred Col-0×Col-0-FTL control is highlighted in green, via the horizontal dashed line. Stars at the top of the plot indicate hybrids with significantly different crossover frequencies to the inbred strain based on chi-square tests. Beneath, the same data are analyzed for the ratios of green (circles, short dashed line) and red (triangles, long dashed line) fluorescent seed to nonfluorescent seed and presented in the same way. c) Sequence similarity dot plots comparing the *CEN178* arrays from a subset of the accessions analyzed in b. Red and blue shading indicate sequence similarity on forward and reverse strands, using a 161 base pair window. Beneath the dotplot, the cM value for the corresponding *CEN5* hybrid from b is shown, which is proportionally shaded. Additionally, beneath the dot plot are text indicators showing which accessions showed segregation distortion in b. d) For the Col-0, Jm-0, and Ler-0 Eurasian accessions, and the Cvi-0, Elk-3, Rab-1, and Tanz-1 relict accessions, the CENH3 ChIP-seq enrichment (upper, purple) and DNA methylation (lower) in CG (dark pink), CHG (pink), and CHH (light pink) contexts across the *CEN1* region are shown. The position of *CEN178* arrays is indicated by ticks on the *x*-axis on the forward (red) or reverse (blue) strand.

We completed our study by crossing the red-encoding FTL T-DNA *CR1136* at base pair position 7,998,504 of chromosome 5 in the Col-CEN genome assembly, and a green-encoding FTL T-DNA *CG358* at position 15,759,366, to generate the *CENTROMERE5* (*CEN5*) interval, which is 7.76 Mb long (Fig. 6a, Supplementary Fig. 1 and Supplementary Table 1) (Wu et al. 2015; Poethig et al. 2022). Relative to previous Col-0×Ler-0 genetic maps, 48.9% (3.8 Mb) of the *CEN5* interval corresponds to NRZ, with the remainder corresponding to LRZ (17.3%, 1.34 Mb) and chromosome arm (33.7%, 2.62 Mb) sequences (Fernandes et al. 2024). Compared to the other *CEN* FTL intervals, *CEN5* contains a higher proportion of pericentromeric and arm sequences, in addition to the *CEN178* satellite arrays (Supplementary Fig. 1). In *CEN5*, 7 hybrids showed significantly higher crossover frequency compared to inbreds, and 2 were significantly lower (chi-square tests, all *P* ≤ 4.87 × 10^−6^) (Fig. 6b and Supplementary Table 7), with the hybrids derived from relicts IP-Alo-19 and IP-Cat-0 showing the highest crossover frequency. These Iberian relict accessions share a similar *CEN178* array, but which is orientated on the opposite strand to the Col-0 array (Fig. 6c). In contrast to the other centromeres, the Cvi-0×Col-0 hybrid had significantly lower crossover frequency (chi-square test, *P* = 1.77 × 10^−46^), suggestive of genetic or epigenetic features in this interval that suppress recombination (Fig. 6, b and c and Supplementary Table 1). While the Cvi-0 *CEN178* array is substantially larger than Col-0, the Tanz-1 array is even larger, and Tanz-1×Col-0 hybrids had significantly higher crossover frequency than Col-0 inbreds (chi-square test, *P* = 5.81 × 10^−10^), which indicates independence of *CEN178* array structural variation on centromere-proximal recombination (Fig. 6, b and c, Supplementary Fig. 9 and Supplementary Table 1). Five hybrids showed significant segregation distortion in favor of the Col-0 chromosome (chi-square tests, all *P* ≤ 0.031), whereas one hybrid, ANGE-B-2×Col-0, showed distortion against Col-0 (chi-square test, *P* = 5.53 × 10^−5^) (Fig. 6b and Supplementary Table 1). As for the other centromeres, variation in centromere-proximal crossover frequency was uncorrelated with the incidence of segregation distortion (Fig. 6, b and c, Supplementary Fig. 2 and Supplementary Table 1). With respect to CENH3 loading, the Rab-1 and Tanz-1 relict accessions both displayed relatively large 5.7 and 6.5 Mb *CEN178* arrays, respectively, that contained broad 5.57 and 7.45 Mb regions of CENH3 enrichment, although these were not associated with consistent effects on crossover frequency, or distorted inheritance (Fig. 6b to d and Supplementary Table 1).

### Centromere-proximal crossover frequency and segregation distortion compared across all 5 chromosomes

**Fig. 7.**
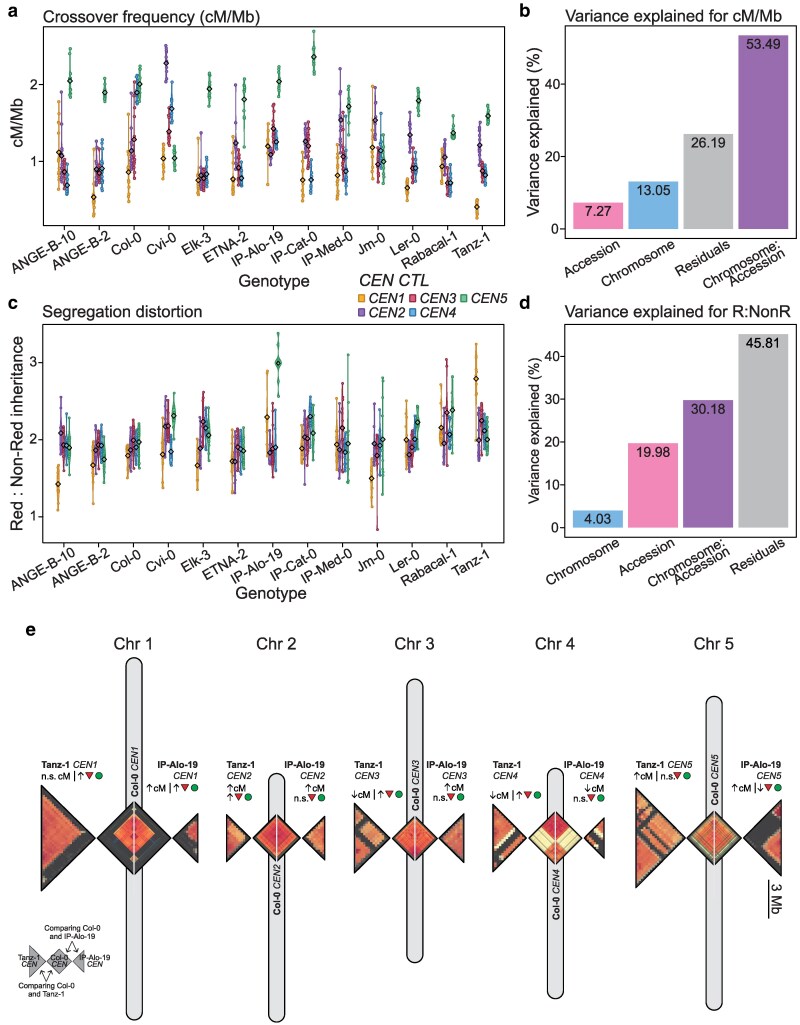
Crossover frequency and segregation distortion across 12 Arabidopsis hybrids relative to Col-0 inbreds. a) Violin plots showing the variation in cM/Mb values for different *CEN* FTL intervals in different accession hybrids, and Col-0 inbreds. b) Linear modeling results showing the % variation in crossover frequency explained by accession (pink), chromosome (blue), the interaction between accession and chromosome (purple), and residuals (grey). c) As for A, but showing variation in red to nonred fluorescence ratios for each *CEN* FTL interval in different accession hybrids, and Col-0 inbreds. d) As for B, but modeling variation in red to nonred fluorescence ratios. e) For each chromosome, a sequence identity heat map is shown for Col-0 compared to Tanz-1 (where 4 of 5 centromeres showed significantly distorted inheritance against Tanz-1) and IP-Alo-19 (where 4 of 5 centromeres showed significantly higher crossover frequency compared to inbreds) centromeres. Text labels indicate whether the Tanz-1 and Alo-19 hybrids showed significantly higher (up arrow), lower (down arrow), or no significant (n.s) change, with the same signifiers used to indicate segregation distortion of the FTL T-DNAs, either in favor of Col-0 (up arrow), against (down arrow), or no significant change (n.s.). Beneath are histograms showing the number of estimates with specific % identity in the sequence identity heat maps for each accession and each centromere.

In total, we quantified crossover frequency and segregation distortion in 60 hybrid combinations derived from 12 accessions that were analyzed across 5 centromeres each and compared them with inbred Col-0 FTL strains (Figs. 2 to 7 and Supplementary Tables 1 to 7). In total, 49 of the hybrid centromere combinations had significantly higher (*n* = 27), or lower (*n* = 22), centromere-proximal crossover frequency, compared to Col-0 inbreds, consistent with widespread modification of centromere-proximal recombination by natural variation (Figs. 2 to 7 and Supplementary Table 1). The most consistently high-recombining hybrids were those derived from the relict accessions Cvi-0 and IP-Alo-19, where 4 of 5 *CEN* intervals showed significantly higher crossover than Col-0 inbreds, in each case (Fig. 7a and Supplementary Table 1). In contrast, crossovers in Ler-0×Col-0 hybrids were suppressed compared to inbreds, across 4 of the 5 centromeres (Fig. 7a and Supplementary Table 1), which together may indicate the action of *trans-*modifying effects in these hybrids that co-ordinately influence recombination on multiple centromeres. Despite being more genetically diverged, the relict accessions produced proportionally more hybrids with significantly higher recombination (23 of 40, 65%) than the Eurasian accessions (4 of 20, 20%) (Supplementary Table 1). This is also true when Iberian (9 of 15, 60%) and non-Iberian (14 of 25, 56%) relicts are considered separately (Supplementary Table 1). Hybrid crossover frequency was most likely to be suppressed within *CEN4*, relative to inbreds, compared to the other centromeric intervals, with reduced crossovers in all but one of the 12 hybrids (Fig. 7a and Supplementary Table 1). Suppression of *CEN4* crossover frequency is almost certainly due to the well-documented knob-inversion in Col-0 that is not present in the other accessions in our study set (Supplementary Fig. 8) (Fransz et al. 2000, 2016). Together, this indicates widespread modification of proximal crossover frequency, although with accession and centromere-specific effects.

Compared to crossover frequency, we observed fewer (18 of 60) instances of significant segregation distortion in the hybrids compared to inbreds (Fig. 7b and Supplementary Table 1). There was no significant correlation between changes to crossover frequency and the incidence of segregation distortion (Supplementary Fig. 2 and Supplementary Table 1), indicating that these phenomena are largely uncoupled. We observed eighteen instances of the Col-0 FTL chromosomes over-transmitting, and 5 cases where there was under-transmission, in hybrids compared to inbreds, with a greater incidence of distortion on the longer chromosomes 1 and 5 (13 of 18) (Fig. 7b and Supplementary Table 1). Proportionally, there was a similar incidence of segregation distortion amongst hybrids with the diverged relict accessions (16 of 40, 40%), compared to hybrids between the Eurasians (7 of 20, 35%) (Fig. 7 and Supplementary Table 1). The Iberian (4 of 15, 27%) and non-Iberian (12 of 25, 48%) relict hybrids both showed distorted inheritance (Supplementary Table 1). Interestingly, the Tanz-1 accession showed distortion in favor of Col-0 across 4 of 5 centromeres, potentially indicating a *trans* modifying effect acting across the centromeres in this accession (Fig. 7, b and c and Supplementary Table 1).

To further consider our data together, we used linear modeling to investigate which factors explain variation in crossover frequency and segregation distortion. First, as each FTL interval varies in physical size, we used crossovers per megabase (cM/Mb) as the response variable. We considered the model:


cM/Mb∼chromosome×accession

where “chromosome” denotes the CTL interval analyzed (*CEN1* to *CEN5*), and “accession” denotes the hybrid background that was measured. Our model explained 85.0% of the variation in crossover frequency, where the interaction between chromosome and accession explained the greatest amount (53.5%), compared to chromosome (13.1%), and accession (7.3%) alone (Fig. 7b and Supplementary Table 8). This indicates that the influence of a specific centromere on crossover frequency is dependent on the accession crossed to. We tested additional models for crossover frequency that included the stepwise addition of the variables *CTL* interval length, gene density, transposon density, and *CEN178* density, but found these did not explain additional variation (Supplementary Table 8). Including segregation distortion data in the model explained a small amount of crossover variation (0.2% for red inheritance and 0.6% for green inheritance), consistent with observations made for individual centromeres. Genotype replicates were included as a random variable to control for experimental replication and were not found to contribute to the variance explained.

We took a similar approach to model genetic control of segregation distortion. For each *CTL* interval, we previously observed that the inheritance ratios of red and green fluorescence were highly correlated (Supplementary Fig. 2), and similar results were obtained modeling the red or green seed fluorescence data (Supplementary Table 8). We considered the model:


segregationdistortion∼chromosome×accession

The models explained 56.4% (green) and 53.2% (red) of the variation in distortion, where the interaction between chromosome and accession explained the greatest amount of variance (green = 35.2%, red = 30.2%), compared to chromosome (green = 2.0%, red = 4.0%) and accession (green = 20.6%, red = 20.0%) alone (Fig. 7d and Supplementary Table 8). This indicates that, like for crossover frequency, the influence of a specific centromere is dependent on the accession crossed to. In contrast to crossover frequency, accession had a stronger individual effect than chromosome (Fig. 7d and Supplementary Table 8). Including cM/Mb data as a variable explained a small amount of variation in distortion (green = 2.24%, red = 1.63%) (Supplementary Table 8), consistent with our previous observations that crossover frequency and distortion are not correlated. In contrast to cM/Mb, including the annotation features FTL interval size, transposon density, gene density, and *CEN178* density explained a greater amount of variation in distortion (green = 69.45%, red = 66.67%) (Supplementary Table 8), potentially indicating a stronger contribution of *cis*-acting genetic variation on distortion compared to crossover frequency.

Finally, as CENH3 is a key determinant of centromere identity (Talbert et al. 2002; McKinley and Cheeseman 2016), we investigated whether genetic polymorphism in CENH3 might contribute to variation in centromere-proximal crossover frequency, or segregation distortion, observed between the hybrids studied here (Supplementary Fig. 10). We observed few amino acid polymorphisms between Col-0 CENH3 and sequences in the accessions crossed to; with a G to A substitution in Elk-3 and IP-Cat-0, and a S to Y substitution in Cvi-0 (Supplementary Fig. 10). In contrast, we observe a high number of amino acid variants, clustered in the N-terminal tail of CENH3, between *A. thaliana* and the sister species *A. lyrata* (Supplementary Fig. 10), consistent with CENH3 diverging rapidly between species in the Arabidopsis genus (Kawabe, Nasuda, and Charlesworth 2006; Maheshwari et al. 2015). We conclude that genetic variation in CENH3 within *A. thaliana* is unlikely to explain the hybrid variation we observe in centromere-proximal crossover frequency and segregation distortion.

## Discussion

We studied 12 Arabidopsis hybrids to survey the effects of naturally occurring variation on meiotic crossover frequency and segregation distortion across all 5 centromeres. We found widespread modification of centromere-proximal crossover frequency, with approximately equal cases where hybrid recombination was higher or lower than observed in inbreds. When considering genetic variation within the FTL intervals, including the size and structure of the *CEN178* satellite arrays and nucleotide diversity, there was no clear pairwise relationship between sequence divergence and recombination frequency. However, our linear modeling indicates a strong interacting effect of *cis* and *trans* genetic variation on crossover frequency. Interestingly, the accessions producing the highest-recombining hybrids, Cvi-0 and IP-Alo-19, are both diverged relict accessions compared to the Eurasian Col-0 reference strain. This indicates that an inhibitory effect of greater *cis* genetic divergence does not easily explain variation in centromere-proximal meiotic recombination levels. These observations are also consistent with previous work, where Arabidopsis hybrids showed higher recombination than inbreds (Barth et al. 2001; Ziolkowski et al. 2015; Blackwell et al. 2020; Dluzewska et al. 2023).

Larger inversions and structural variants in the chromosome arms are well known to suppress crossover recombination in *cis* (Sturtevant 1917), and this is also the case for the 1.2 Mb knob-inversion polymorphism within *CEN4* of Col-0 (Fransz et al. 2000, 2016). As Cvi-0×Col-0 and IP-Alo-19×Col-0 hybrids had high recombination rates across 4 of 5 centromeres, while recombination was low in 4 of the centromeres in Ler-0×Col-0 hybrids, we propose that Cvi-0, IP-Alo-19, and Ler-0 may also contribute *trans*-acting recombination-modifying loci that exert a global effect on centromere-proximal crossovers across multiple chromosomes. Further quantitative trait loci mapping experiments using populations derived from these hybrids may reveal new genes that regulate the levels of centromere-proximal crossover frequency, and the extent of *cis* vs *trans* acting variation on recombination. We did not observe a strong effect of structural variation between the centromere satellite arrays on crossover frequency. However, as centromere-proximal crossovers have been fine-mapped to pericentromeric “low-recombining zones” (LRZ) that flank the central “nonrecombining zone” (NRZ) that contains the satellite arrays (Fernandes et al. 2024), this may effectively shield recombination rates across these regions from the effect of structural variation in the core centromere regions.

Segregation distortion can be produced by a variety of causes during meiosis, or post-meiosis during germ line development. As our experiments involved self-fertilization of Arabidopsis, we are measuring both male and female meiotic transmission simultaneously. Meiotic drive of centromeres has been detected in female meiosis of mice, as well as monkeyflowers, where only one of the meiotic products survives to produce the egg cell (Fishman and Willis 2005; Fishman and Saunders 2008; Malik 2009; Chmátal et al. 2014; Akera et al. 2017, 2019; Iwata-Otsubo et al. 2017; Lampson and Black 2017; Kursel and Malik 2018; Kumon et al. 2021; Finseth et al. 2022). This pattern of development is similar in Arabidopsis ovules, where the megasporocyte gives rise to a tetrahedral or decussate tetrad, and where the most chalazal cell enlarges and the remaining 3 degenerate (Webb and Gunning 1990; Bowman 2012), meaning there is an opportunity for female meiotic drive to cause segregation distortion. Asymmetries in the spindle, or cell division, during female meiosis have the potential to be exploited by variant centromeres to cause segregation distortion into the surviving meiocyte (Malik 2009; Chmátal et al. 2014; Akera et al. 2017, 2019; Iwata-Otsubo et al. 2017; Lampson and Black 2017; Kursel and Malik 2018; Kumon et al. 2021). For example, in *Mimulus* interspecific hybrids, a 98:2 centromeric transmission advantage of the *D* haplotype has been observed through female meiosis, and 58:42 drive in con-specific crosses (Fishman and Saunders 2008; Fishman and Kelly 2015). This correlates with a larger cytogenetic fluorescent *in situ* hybridization (FISH) signal for the Cent728 satellite repeats in the driving *D* haplotype (Fishman and Saunders 2008). Similarly, *Mus musculus* hybrids between laboratory strains and wild strains with smaller centromeres (fewer minor satellite repeats and lower CENP-A loading) show biased segregation of the lab-strain centromeres into the egg (Akera et al. 2017, 2019). In our study, although less frequent than centromere-proximal crossover modification, we observed multiple instances of segregation distortion across the 5 centromeres. Although we did not observe a clear pairwise correlation between genetic divergence or the relative sizes of hybrid *CEN178* arrays, our linear modeling indicates a strong combined effect of *cis* and *trans* genetic variation on distortion. We also note several cases such as Tanz-1 *CEN1* and *CEN2* and Rab-1 *CEN4*, where the smaller Col-0 *CEN178* arrays show distorted inheritance at the expense of the larger relict arrays. Therefore, larger satellite arrays may not universally cause a stronger drive in their own favor across species.

As we did not detect a strong correlation between segregation distortion and the size of the *CEN178* satellite arrays or the region of CENH3 loading, this may indicate that other features of centromere organization are important for distorted inheritance. For example, the relative placement of CENH3 regions on the recombining homologous chromosomes during meiosis may be more relevant for distortion, rather than satellite array sizes, or the levels of CENH3 per se. During meiosis, formation of DSBs is more frequent in the chromosome arms and are more likely to initiate interhomolog strand invasion and recombination earlier in prophase-I, due to telomere-led synapsis (Choi et al. 2018; Lambing et al. 2020). Since the Arabidopsis chromosome arms tend to be highly syntenic between accessions (Wlodzimierz et al. 2023a; Lian et al. 2024), they will be more likely to synapse rapidly. We propose that the structurally polymorphic centromeres, which will form fewer DSBs, are likely to synapse in a delayed manner. In this scenario, the flanking syntenic chromosome arms will have aligned and synapsed well, meaning there may be megabase regions in the centre that are not well aligned or synapsed, which could potentially manifest as a synapsis “buckle” composed of the structurally polymorphic regions. Within these potentially buckled centromeric regions, although a similar area may be CENH3-occupied on both homologs, these regions could be positioned differently relative to the spindle microtubules, due to centromere structural polymorphism, in the context of the heterozygous bivalent structure (Supplementary Fig. 11). We speculate that such offsets of CENH3 loading, and their differential interaction with putative spindle asymmetries during female meiosis, may contribute to centromeric segregation distortion that we observe in Arabidopsis hybrids. We also note that multiple instances of segregation distortion have been reported in Arabidopsis, suggesting such interactions could be widespread and result from interactions between multiple *cis* and *trans*-acting factors (Törjék et al. 2006; Salomé et al. 2012; Seymour et al. 2019).

Future studies that explore the cell biology of Arabidopsis meiosis, spindle formation, and how hybrid centromeres interact during meiotic pairing and recombination may reveal mechanisms associated with segregation distortion. It will also be important to consider alternative models that may explain distortion, for example male-based gene drive via spore-killing, involving toxin-antidote systems that are linked to the centromere (Nuckolls et al. 2017; Berube et al. 2024). In conclusion, our study reveals specific hybrid combinations where segregation distortion is evident across each of the Arabidopsis centromeres, as well as variation in meiotic crossover frequency. These hybrids will be useful study systems in which to investigate the molecular basis of segregation distortion and crossover variation over the centromeres.

## Supplementary Material

iyaf264_Supplementary_Data

iyaf264_Peer_Review_History

## Data Availability

Long-read sequencing data and corresponding genome assemblies generated in this study for accessions Jm-0 (GCA_‪977880165) and Elk-3 (GCA_‪977880155) are available at the European Nucleotide Archive (ENA; https://www.ebi.ac.uk/ena/browser/home) under accession number PRJEB83900. Previously released long-read data and assemblies for the remaining accessions are available at ENA under accession number PRJEB55353 (Wlodzimierz et al. 2023a). ChIP-seq reads for Rab-1, Elk-3, and Jm-0 are available under ArrayExpress accession E-MTAB-15384. Supplemental material available at GENETICS online.
