# Supplementary material for: Natural variation modifies centromere-proximal meiotic crossover frequency and segregation distortion in Arabidopsis thaliana
Source: Genetics. 2025 Dec 8;233(4):iyaf264. doi: 10.1093/genetics/iyaf264 (PMC13439696; doi:10.1093/genetics/iyaf264)
Supplement: Supplementary file 1 [file iyaf264_peer_review_history.pdf]

GENETICS

Natural variation modifies centromere-proximal meiotic crossover frequency and segregation distortion in Arabidopsis thaliana

Nicola Gorringe, Stephanie Topp, Robin Burns, Sota Yamaguchi, Fernando Rabanal, Joiselle Fernandes, Detlef Weigel, Tetsuji Kakutani, Matthew Naish, and Ian Henderson

NOTE: The reviews and decision letters are unedited and appear as submitted by the reviewers.
In extremely rare instances and as determined by a Senior Editor or the EIC, portions of a review may be redacted. If a review is signed, the reviewer has agreed to no longer remain anonymous.
The review history appears in chronological order.

Review Timeline:

Submission Date:	2025-07-30
Editorial Decision:	2025-09-04
Resubmission Received:	2025-10-28
Accepted:	2025-11-17

1st Editorial Decision: September 4, 2025

September 1, 2025

GENETICS-2025-308431
Natural variation modifies centromere-proximal meiotic crossover frequency and segregation distortion in Arabidopsis thaliana

Dear Ian:

Two experts in the field have reviewed your manuscript. They both see the value of the results for the field, but they also have suggestions for improving the presentation and tempering the conclusions. While your manuscript is not currently acceptable for publication in GENETICS, we would welcome a substantially revised manuscript. Both reviewers have comments and concerns to be addressed in a revised manuscript. You can read their reviews at the end of this email. We look forward to receiving your revised manuscript. Please let the editorial office know approximately how long you expect to need for revisions.

Upon resubmission, please include:
1. A clean version of your manuscript;
2. A marked version of your manuscript in which you highlight significant revisions carried out in response to the major points raised by the editor/reviewers (track changes is acceptable if preferred);
3. A detailed response to the editor's/reviewers' feedback and to the concerns listed above. Please reference line numbers in this response to aid the editor and reviewers.

Your paper will likely be sent back out for review.

Additionally, please ensure that your resubmission is formatted for GENETICS
https://academic.oup.com/genetics/pages/general-instructions

Follow this link to submit the revised manuscript: Link Not Available

Sincerely,

James Birchler
Associate Editor
GENETICS

Approved by:
Amanda Larracuente
Senior Editor
GENETICS

Reviewer #1 :

The focus of the manuscript is examining how centromere structural variation impacts proximal crossover (CO) frequency and segregation distortion in Arabidopsis thaliana. The centromere and pericentromeric regions around the centromere experience CO suppression. The authors used 12 F1 hybrids to determine if differences in the natural variation of the centromeres affect CO formation. The authors also used fluorescent reporters to score CO frequency and examine segregation distortion. They also examined DNA methylation, genetic polymorphisms, CEN178 satellite arrays, and nucleotide diversity to try to explain CO frequency near centromeres and segregation distortion differences from the inbred Col-0 reference strain.

The study provides value to the community by focusing on centromere-proximal COs, a feature unique to Arabidopsis. Other organisms, such as maize, mice, yeast experience a higher CO frequency in the sub-telomeric regions of the chromosomes. Researchers suspect this difference in CO distribution in Arabidopsis could be due to several factors, such as small genome size, gene density, centromere composition and structure, chromatin environment, and polymorphisms. This study highlights centromere natural variation.

Overall, I like the idea of this story, but the manuscript needs to be rewritten with a purpose and to be concise and clear. I believe the conclusions need to be more clear and more explicit. Most of the conclusions are that there is no significance between these two events, such as the relative sizes of hybrid CEN178 arrays and the incidence of distortion for example (561). The authors overall conclusion (594-598) of segregation distortion being evident and that there is variation in CO frequency is too broad of a statement. Segregation distortion does occur, but not very frequently with being observed 18 out of 60 times (485).

248-508: The manuscript is very repetitive. Is it necessary to have each centromere in the main paper (especially the ones with no correlation to recombination patterns, such as on lines 285-289, 306-308, 313-314, 331-333 for CEN1)? Can some of the centromeres be in the supplemental materials? Even if all five centromeres are retained, I believe the authors could presented their findings in a more effective manner. A potential idea would be to break up each section by topic, such as CO frequency, instead of by centromere. I keep finding myself flipping through to compare each centromere. The manuscript does have a section discussing all five centromeres together, but it is not very organized, clear, or thorough.

74-89: I can understand why the authors start out with the recombination pathway because it is what we know, but these steps are not that important for understanding the study. If the authors keep the recombination pathway overview, it needs to be written from a centromere perspective.

91-106: The authors discuss what influences CO frequency and distribution; however, the topics they highlight are not directly tied to the focus of their study on the impact of centromeres. They are examining sequence polymorphisms, nucleotide diversity, DNA methylation, but they do not address how these features might specifically impact CO frequency and distribution in the centromeric context. Clarifying these connections would make the introduction more relevant.

108-125: The authors discuss what hinders COs, which includes sequence polymorphisms, DNA methylation, and H3K9me2. Given this context, it would be valuable for them to examine H3K9me2 in their study as well. The paragraph appears somewhat fragmented, presenting a collection of facts rather than a focused narrative.

127: It would strengthen the manuscript if the authors explicitly addressed the limitations of using H3 as a marker for defining centromeres, and explained how their chosen approach provides a more accurate definition.

131: The authors should explain what "densely DNA methylated" means, such as how this would impact CO formation or segregation distortion to make it more meaningful/clear to a diverse group of readers.

143-160: The authors talk about how different the centromeres are from each other, but I feel like they could present a better assessment of the centromere composition/diversity. They do examine nucleotide diversity later in the manuscript, but they could provide a stronger case early on.

171-172: The authors need to explain why they are only evaluating epigenetic patterns with a subset of 7 accessions and not all the 12 accessions. They also do not list what the accessions they have chosen are.

Another piece of information the author should add is the current CO rate in the centromere and pericentromeric regions in the control strain Col-0.

184-205: The authors use two FTL T-DNAs markers per chromosome and so they can only detect COs in the interval between the two markers. The FTL CEN intervals have various amounts of the pericentromeric regions. Can the authors add how this might impact the results. The authors keep using "centromere-proximal," can they define "proximal"?

189: The authors should change "FTL" to "fluorescent-tagged locus (FTL)" for the first instance.

207-208: Since the authors do not directly examine at inheritance, they should remove those words and just use "quantify segregation distortion."

225-227: Please point out that the control, Col-0, is part of the Eurasian genetic group.

240: Does this mean that the analysis was actually conducted on the F2 and not F1 generation?

272-273: The authors should provide greater specificity by clarifying what the "size difference" is referring to. This is also the first mention of "size difference" and the authors should add a sentence explaining why they are examining it and how it relates to CO frequency and segregation distortion.

281-289: It would be good if the authors to explained why they think nucleotide diversity would correlate with CO frequency. Nucleotide diversity tends to be sensitive to rare and common variants. I don't know if this would change the results, but have the authors thought about splitting the FTL interval region into rare and common variants to determine if this would provide a significant difference. Nucleotide diversity alone may not be sensitive enough. They could also add θw and Tajima's D in sliding windows to give a stronger assessment of the genetic diversity of the FTL interval region.

291: The authors could add an opening sentence to have a smoother transition.

390-392: The authors should clarifying what is being over- or under-transmitted.

376-429: The authors discuss CO frequency, segregation distortion, satellite array polymorphisms, centromeric structural polymorphisms, nucleotide diversity, and chromatin or epigenetic organization for centromere 1, 2 & 5 in more detail, but were incomplete for centromeres 3 and 4. The authors need to have this information for all the chromosomes. Specifically, the segregation distortion part was not as clear for centromere 3 &4.

420-422: Do the other accessions share the knob?

465-466: What are the sixty-hybrid combination? Can the authors introduce them and explain what they are and mean. Until this point I thought the authors were examining only 12 other accessions.

471-478: The CO frequency is discussed for the relict accessions. The Eurasian accessions are mentioned, but the non-Iberian relict accessions are not brought up. The authors need to add more details or explain why they are not looking at non-Iberian relict accessions.

476: The authors mention trans-modifying effects and the large knob in chromomere 4 for different CO frequences among the 12 lines, but they don't state what trans-modifying effects could specifically be and which hybrids have this knob.

476-484: The authors say the relict accessions, which are more diverse, have higher recombination rates, but when polymorphisms are discusses, there is never a correlation. The authors need provide an alternative reason for this result.

Also, the COs around CEN4 is suppressed and it is discussed why, but the COs around CEN3 being suppressed is not discussed.

489-490: What is being over-transmitted and under transmitted?

490-498: These lines should be rewritten to be clearer.

465-498: This paragraph currently reads as a collection of loosely connected ideas, which makes it difficult to follow. I recommend revising it into a clearer narrative. Consider breaking it into multiple paragraphs, with transition sentences to guide the reader through the logic.

502-503: The discussion of amino acid polymorphisms feels abrupt at this stage of the manuscript. If the authors intend to highlight these results, they should introduce earlier why amino acid polymorphisms are relevant to their study. This would provide better context and ensure the section integrates smoothly into the overall flow of the paper.

504-505: The authors should explain why they are using A. lyrata. Why is variation at the N-terminal tail of CENH3 significant? What kind of variation are the authors referring to?

500-508: The conclusion presented in this paragraph is potentially valuable, but the connection between the evidence provided and the conclusion is unclear. I recommend clarifying how the data or arguments in the paragraph support this point so the reader can clearly follow the reasoning.

527-540: This paragraph begins with large inversions and structural variants and then switches to QTLs. Can the authors add a smoother transition and add the relevance/connection.

540-542: The transition into the discussion of meiotic drive in female meiosis feels abrupt. Since the study examines seeds rather than pollen, both female and male meiosis are being evaluated. Highlighting this connection could provide a reason for discussing this topic.

546: Can the authors expand on how this is similar in Arabidopsis?

554: The authors should spell out "FISH".

550-557: Can the authors explain the points of these two studies in relation to their study? How is the transmission ratio relevant to their case? For the second study, are they trying to point out the centromere size or that their lab strains are not reliable? Please add some clarity.

566-587: For segregation distortion, the authors provide a good potential reason, but please further explain the "synapsis buckle".

F1A: Please add a measure of diversity for each centromere.

F1B: Please add which strains are in which genetic group?

F2-6C: All the strains analyzed in this panel are different for each centromere and for each part of the figure. To try to figure out why, I examined the strains in part B that are significant to see if those are the same ones examined at in part C, but it doesn't hold. These are also not the same combination of strains examined at in part D, the subset to examine epigenetic organization. While the bar plot at the bottom in part C (CO frequency and segregation distortion) is clear and useful, the overall presentation of part C is confusing. The author needs a clearer way to convey this information.

FS1: For the "DNA methylation" legend, the colors appear to be dark red, pink, light pink, instead of dark red, orange, and yellow. For the "features" legend, the "TEs" annotation appears to be orange, and not green. Updating the legend to reflect these colors would improve clarity.

FS3: These plots are very small I can barely read them. There are parts A-E, but in the description, there are only parts A-D. The plots could be organized better to reduce the white space.

957: Please state what TRASH is.

959: The correlation line looks more black than blue.

FS4 & FS5: These figures seem out of order. Maybe FS4 can be switched so all the same graphs are next to each other.

Reviewer #2 :

This is an interesting work that studies meiotic crossover around the centromeric region, as well as meiotic segregation distortion using fluorescent seed markers. Twelve Arabidopsis accessions, representing a broad range of ecogeographical diversity, and whose genomes and centromeric arrays were sequenced and fully assembled, were crossed to testers in the Col-0 background, one tester for each chromosome. Then, the authors attempted to correlate the crossover and segregation distortion to sequence or structural polymorphism, to epigenetic patterns, or to CENH3 variants. So we are having in total 60 segregating populations of F2 seeds (12 parental lines x 5 testers in Col-0), and for each combination, the correlation with genetic and epigenetic parameters was analyzed. While the reading was tough because of the abundance of details, overall, the work was well done. 12 Replicas were made for each hybrid, and a large number of seeds were counted, so that the statistics of crossover rate or segregation distortion measurements are solid.
I think that the results are important, and the discussion is fine. However, I would request that the authors revise their conclusions, in particular regarding the title, which is very vague and doesn't say much, and the abstracts, in a more direct/candid manner. What I mean is that it seems that the authors are trying hard to say something positive about occasional trends, when in fact, it seems that each centromere in each cross is a different story, and that no clear conclusions can be drawn, and if there are some trends sometimes, they are weak, and they were not subjected to a statistical analysis. The author should embrace this complexity. In fact, I found that the strength of the work is in providing a strong insight into what DOES NOT explain crossover frequencies around the centromere or what DOES NOT explain segregation distortion. Ruling out possible mechanisms is very important for future work by others.
The main conclusion is that the size of the CENH3-enriched arrays, the sequence polymorphism of the region between the markers, and the patterns of methylation DID NOT correlate with crossover rates or with segregation distortion. Most effects were accession or centromere-specific. Moreover, there was NO correlation between centromere-proximal crossover and segregation distortion. These conclusions should be clear from the abstract and the title.

Associate Editor Comments:

GENETICS-2025-308431
Natural variation modifies centromere-proximal meiotic crossover frequency and segregation distortion in Arabidopsis thaliana

Response to reviewers

We thank the reviewers for their helpful and insightful suggestions to improve our manuscript. We include a point-by-point response to their comments below. The reviewer's comments are highlighted in **bold**, with our response in blue text.

Reviewer #1:

The focus of the manuscript is examining how centromere structural variation impacts proximal crossover (CO) frequency and segregation distortion in Arabidopsis thaliana. The centromere and pericentromeric regions around the centromere experience CO suppression. The authors used 12 F1 hybrids to determine if differences in the natural variation of the centromeres affect CO formation. The authors also used fluorescent reporters to score CO frequency and examine segregation distortion. They also examined DNA methylation, genetic polymorphisms, CEN178 satellite arrays, and nucleotide diversity to try to explain CO frequency near centromeres and segregation distortion differences from the inbred Col-0 reference strain.

The study provides value to the community by focusing on centromere-proximal COs, a feature unique to Arabidopsis. Other organisms, such as maize, mice, yeast experience a higher CO frequency in the sub-telomeric regions of the chromosomes. Researchers suspect this difference in CO distribution in Arabidopsis could be due to several factors, such as small genome size, gene density, centromere composition and structure, chromatin environment, and polymorphisms. This study highlights centromere natural variation.

Overall, I like the idea of this story, but the manuscript needs to be rewritten with a purpose and to be concise and clear. I believe the conclusions need to be more clear and more explicit. Most of the conclusions are that there is no significance between these two events, such as the relative sizes of hybrid CEN178 arrays and the incidence of distortion for example (561). The authors overall conclusion (594-598) of segregation distortion being evident and that there is variation in CO frequency is too broad of a statement. Segregation distortion does occur, but not very frequently with being observed 18 out of 60 times (485).

248-508: The manuscript is very repetitive. Is it necessary to have each centromere in the main paper (especially the ones with no correlation to recombination patterns, such as on lines 285-289, 306-308, 313-314, 331-333 for CEN1)? Can some of the centromeres be in the supplemental materials? Even if all five centromeres are retained, I believe the authors could presented their findings in a more effective manner. A potential idea would be to break up each section by topic, such as CO frequency, instead of by centromere. I keep finding myself flipping through to compare each centromere. The manuscript does have a section discussing all five centromeres together, but it is not very organized, clear, or thorough.

We appreciate that the findings of our study are complex. To further consider the totality of our data in a single analysis, we used linear modelling to investigate which factors explain variation in centromere-proximal crossover frequency and segregation distortion.

First, as each FTL interval varies in physical size, we used crossovers per megabase (cM/Mb) as the response variable. We considered the model:

$$cM/Mb \sim chromosome * accession$$

Where 'chromosome' denotes the CTL interval analysed (*CEN1* to *CEN5*), and 'accession' denotes the hybrid background that was measured. Our model explained 85.01% of the variation in crossover frequency, where the interaction between chromosome and accession explained the greatest amount (53.49%), compared to chromosome (13.05%), and accession (7.27%) alone. This indicates that the influence of a specific centromere on crossover frequency is dependent on the accession crossed to.

We tested additional models for crossover frequency that included the stepwise addition of the variables *CTL* interval length, gene density, transposon density, and *CEN178* density, but found these did not explain additional variation.

Further, including segregation distortion data in the model explained a small amount of crossover variation (0.15% for red inheritance and 0.59% for green inheritance), consistent with our observations made for individual centromeres where the incidence of changes to crossover and distortion did not correlate. Genotype replicates were included as a random variable to control for experimental replication and were not found to contribute to the variance explained.

We took a similar approach to model genetic control of segregation distortion. For each *CTL* interval, we previously observed that the inheritance ratios of red and green fluorescence were highly correlated, and similar results were obtained modelling the red or green seed fluorescence data. We considered the model:

segregation distortion ~ chromosome * accession

These models explained 56.36% (green) and 53.22% (red) of the variation in distortion, where the interaction between chromosome and accession explained the greatest amount of variance (green=35.19%, red=30.18%), compared to chromosome (green=1.99%, red=4.03%) and accession (green=20.58%, red=19.98%) alone. This indicates that, like for crossover frequency, the influence of a specific centromere is dependent on the accession crossed to.

In contrast to crossover frequency, accession had a stronger individual effect than chromosome. Including cM/Mb data as a variable explained a small amount of variation in distortion (green=2.24%, red=1.63%), consistent with our previous observations that crossover frequency and distortion are not correlated.

In contrast to the crossover frequency model, including the annotation features FTL interval size, transposon density, gene density and *CEN178* density explained a greater amount of variation in distortion (green=69.45%, red=66.67%), potentially indicating a stronger contribution of *cis*-acting genetic variation on distortion, compared to crossover frequency.

These analyses are now presented in the final section of the results, in Fig. 7A-7D, and Table S8. Fig S2, S3, and Table S1 also provide data and analysis across all five centromeres.

74-89: I can understand why the authors start out with the recombination pathway because it is what we know, but these steps are not that important for understanding the study. If the authors keep the recombination pathway overview, it needs to be written from a centromere perspective.

Our study examines crossover recombination and distortion, and so we feel it is necessary to generally introduce meiosis. To make the first paragraph more relevant to the study, we briefly explain centromere behaviour in meiosis-I and meiosis-II and provided a reference (page 4).

We feel the subsequent consideration of variation in recombination frequency is also required, including occurrence of crossover hot spots and cold spots, as centromeres are well known to be crossover cold spots in diverse species.

91-106: The authors discuss what influences CO frequency and distribution; however, the topics they highlight are not directly tied to the focus of their study on the impact of centromeres. They are examining sequence polymorphisms, nucleotide diversity, DNA methylation, but they do not address how these features might specifically impact CO frequency and distribution in the centromeric context. Clarifying these connections would make the introduction more relevant.
In paragraph 3 of the Introduction, we address how structural variation and heterochromatic modifications are known to impact meiotic recombination, which is directly relevant to the study and is relevant material to set the context.

108-125: The authors discuss what hinders COs, which includes sequence polymorphisms, DNA methylation, and H3K9me2. Given this context, it would be valuable for them to examine H3K9me2 in their study as well. The paragraph appears somewhat fragmented, presenting a collection of facts rather than a focused narrative.

H3K9me2, a well-studied heterochromatic mark in Arabidopsis, and is known to strongly correlate with DNA methylation [PMID: 34762468], and both H3K9me2 and DNA methylation were found to be associated with suppressed crossovers when ectopically added to an Arabidopsis recombination hot spot [PMID: 26494791]. As H3K9me2 correlates strongly with DNA methylation, we feel it would be redundant to generate H3K9me2 data across the accessions studied. However, as we have previously generated H3K9me2 ChIP-seq data from the Col-0 accession [PMID: 32024691], we now incorporate these data into Figure S1 where landscapes of DNA methylation and other genome features are shown along the Col-0 chromosomes together with the position of the FTL intervals used.

127: It would strengthen the manuscript if the authors explicitly addressed the limitations of using H3 as a marker for defining centromeres, and explained how their chosen approach provides a more accurate definition.

The functional definition of centromeres, as the zone of CENH3 or CENP-A loading, is well accepted in the field [e.g. PMID: 26601620]. With respect to our study, it is relevant that while the Arabidopsis satellite arrays may be greater than 6 Mb, typically a smaller ~ 1-2 Mb region is CENH3-occupied [PMID: 37198485]. As CENH3 directly assembles the kinetochore, which mediates microtubule attachment, the CENH3-occupied region is typically defined as the centromere proper or functional centromere; rather than the totality of satellites, some of which are unoccupied. We have added text to this paragraph to clarify this definition.

131: The authors should explain what "densely DNA methylated" means, such as how this would impact CO formation or segregation distortion to make it more meaningful/clear to a diverse group of readers.

The extent and distribution of DNA methylation across Arabidopsis centromeres have been reported in multiple previous studies [e.g. PMID:38254210, PMID:34762468]. We also provide maps of DNA methylation levels in Col-0 in Figure S1, and in panel D of Figures 2-6 for a subset of the accessions studied.

We and others have previously shown that loss of CG and non-CG context DNA methylation impact centromere-proximal crossover frequency, although with opposite effects. Specifically, CG mutants such as *ddm1* and *met1* decrease centromere-proximal crossovers, whereas non-CG mutants increase them [PMID:38254210, PMID:26494791, PMID:22876192, PMID:29530927]. We have added text and citations to the Introduction (page 6) to discuss these findings. How DNA methylation loss would influence segregation distortion has not yet been investigated. However, some of the hybrids we report in this study could be interesting to use in follow-up experiments, which we highlight in Discussion section.

143-160: The authors talk about how different the centromeres are from each other, but I feel like they could present a better assessment of the centromere composition/diversity. They do examine nucleotide diversity later in the manuscript, but they could provide a stronger case early on.

Previously, we analysed the FTL intervals in terms of their size in Col-0 versus their size in the accession crossed to, nucleotide diversity, as well as the difference in *CEN178* satellite copy number between the accessions. To extend this analysis we have now generated gene and transposon annotation in each accession genome studied, using Helixer and EDTA, respectively. We tested whether these features correlate with crossover frequency, or segregation distortion, on a per chromosome basis, which they do not. We also included these features as variables in our linear modelling and found they did not explain additional variation in crossover frequency. We have added the new gene and transposon annotation data to Table S1, and included scatterplots in Fig. S3, and in discussion of our modelling results.

171-172: The authors need to explain why they are only evaluating epigenetic patterns with a subset of 7 accessions and not all the 12 accessions. They also do not list what the accessions they have chosen are.

We did not perform ChIP-seq and DNA methylation mapping in each accession due to resource and time limitations. As the text lines referred to by the reviewer are located at the end of the Introduction, we didn't feel that adding a list of accessions here was useful. Instead, we have noted that the seven accessions analysed for chromatin represent both Eurasian and relict diversity. Detailed information on the accessions chosen, including their geographic origin, are provided in the first results section, and in Tables S1 and S2.

Another piece of information the author should add is the current CO rate in the centromere and pericentromeric regions in the control strain Col-0. 184-205: The authors use two FTL T-DNAs markers per chromosome and so they can only detect COs in the interval between the two markers. The FTL CEN intervals have various amounts of the pericentromeric regions. Can the authors add how this might impact the results. The authors keep using "centromere-proximal," can they define "proximal"?

For each centromere we provide FTL crossover frequency measurements in Col-0 inbreds. To provide a more fine-grained map of crossover frequency within the FTL intervals, it is necessary to employ crosses with intervening polymorphic genetic markers. For example, we previously reported a deep genetic map from a Col-0 x Ler-0 cross and analysed the recombination landscape around the centromeres [Fernandes et al., PMID:38254210]. For example, we report the chromosome arm average crossover frequency from this cross, which was 3.8 cM/Mb. In the Fernandes et al study, we defined a central non-recombining zone (NRZ) where we never observed a crossover for each centromere. The NRZs are flanked by 1 cM regions that we termed low-recombining zones (LRZs), where recombination is suppressed compared to the chromosome arms. The remainder of the chromosomes were defined as arm regions. For each FTL interval, we now provide a quantification for what fraction of the interval corresponds to NRZ, LRZ, or chromosome arm sequence, as well as providing the associated physical distances. Our use of 'proximal' here means adjacent or close to the centromere.

189: The authors should change "FTL" to "fluorescent-tagged locus (FTL)" for the first instance.

Thank you, we have spelled this out on first usage.

207-208: Since the authors do not directly examine at inheritance, they should remove those words and just use "quantify segregation distortion."

We have made the suggested change.

225-227: Please point out that the control, Col-0, is part of the Eurasian genetic group.

We have added this information.

240: Does this mean that the analysis was actually conducted on the F2 and not F1 generation?

The analysis was performed on the F_2 seed, but in effect, we are analysing recombination events that happened during meiosis of the F_1 parent.

272-273: The authors should provide greater specificity by clarifying what the "size difference" is referring to. This is also the first mention of "size difference" and the authors should add a sentence explaining why they are examining it and how it relates to CO frequency and segregation distortion.

The size difference referred to is the difference in the number of centromeric *CEN178* satellite repeat copies between the two accessions studied. We have added a sentence to make this definition clear.

281-289: It would be good if the authors to explained why they think nucleotide diversity would correlate with CO frequency. Nucleotide diversity tends to be sensitive to rare and common variants. I don't know if this would change the results, but have the authors thought about splitting the FTL interval region into rare and common variants to determine if this would provide a significant difference. Nucleotide diversity alone may not be sensitive enough. They could also add θw and Tajima's D in sliding windows to give a stronger assessment of the genetic diversity of the FTL interval region.

We would like to clarify that in this paper we used pi only in the context of the crosses that we made. We are calculated pairwise nucleotide divergence within the FTL intervals between each accession crossed to Col-0, not as a population-level measure of nucleotide diversity. Our aim here is to test whether nucleotide divergence within the FTL regions can help explain variation in recombination or segregation distortion. We are not discussing the allele frequencies within natural populations of Arabidopsis.

Pi is more informative than θw in our case, which in this context would simply be the total number of SNPs between the accession crossed to and Col-0. This metric is not as informative as pi, as it excludes non-variant sites. Tajima's *D* is also not informative here as we are not testing for neutrality on population-level polymorphisms. Therefore, we do not believe that these statistics would add to our analysis.

291: The authors could add an opening sentence to have a smoother transition.

We have changed this sentence to provide a better introduction to the section.

390-392: The authors should clarifying what is being over- or under-transmitted.

We have attempted to clarify this sentence.

376-429: The authors discuss CO frequency, segregation distortion, satellite array polymorphisms, centromeric structural polymorphisms, nucleotide diversity, and chromatin or epigenetic organization for centromere 1, 2 & 5 in more detail, but were incomplete for centromeres 3 and 4. The authors need to have this information for all the chromosomes. Specifically, the segregation distortion part was not as clear for centromere 3 & 4.

We have modified the text to make sure the same information is presented and discussed for each centromere.

420-422: Do the other accessions share the knob?

Only the Col-0 accession carries the knob inversion. In the results section, we state that "Col-0 is documented to have a large 'knob' inversion on the short arm of chromosome four that spans the *CR909* FTL T-DNA (Fransz *et al.* 2000, 2016), which is not shared by any of the twelve parental accessions crossed to". This is also shown by SyRi comparisons in Figure S8.

465-466: What are the sixty-hybrid combination? Can the authors introduce them and explain what they are and mean. Until this point I thought the authors were examining only 12 other accessions.

The sixty combinations emerge from twelve hybrids x five *CEN* FTL interval = sixty combinations analysed in total.

471-478: The CO frequency is discussed for the relict accessions. The Eurasian accessions are mentioned, but the non-Iberian relict accessions are not brought up. The authors need to add more details or explain why they are not looking at non-Iberian relict accessions.

Thank you for this suggestion. We have now additionally analysed the Iberian and non-Iberian relicts separately and reported the results.

476: The authors mention trans-modifying effects and the large knob in chromomere 4 for

different CO frequences among the 12 lines, but they don't state what trans-modifying effects could specifically be and which hybrids have this knob.

The knob inversion is unique to the Col-0 reference strain in the set of accessions we analysed. We have added this information to the text. We do not have conclusive evidence for trans acting modifiers; but we infer their possible action as in some crosses four of five FTL intervals showed significantly higher, or lower, recombination compared to inbreds, with the same direction of change (Cvi-0, Alo-19 and Ler-0).

Further, from our linear modelling, the interaction between accession and chromosome can explain 85% of variation in crossover frequency, and 53-56% of variation in segregation distortion. We propose that chromosome and accession effects are likely explained by *cis* and *trans* acting genetic variation, respectively. We highlight in the Discussion that future genetic mapping approaches in these crosses may therefore identify novel regulators of centromere recombination, or inheritance.

476-484: The authors say the relict accessions, which are more diverse, have higher recombination rates, but when polymorphisms are discusses, there is never a correlation. The authors need provide an alternative reason for this result.

Our modelling results indicate that 85% of variation in crossover frequency can be explained by the effects of chromosome and accession, and their interaction. We interpret this as indicating that *cis* and *trans* acting variation combine to influence crossover frequency.

Also, the COs around CEN4 is suppressed and it is discussed why, but the COs around CEN3 being suppressed is not discussed.

All centromeres, not just *CEN3* and *CEN4*, showed hybrid combinations that were significantly lower than Col-0 inbreds. Due to the large knob inversion being present only in the Col-0 FTL reference accession, we propose that this causes the majority of *CEN4* hybrids to show significantly lower crossover frequency, compared to inbreds. We are not currently able to explain the other suppressed hybrid recombination patterns in terms of specific patterns of structural variation. Therefore, we have modified this section to focus specifically on *CEN4*.

489-490: What is being over-transmitted and under transmitted?

The sentence clearly states that 'Col-0 FTL chromosomes' are what is being over- or under-transmitted.

490-498: These lines should be rewritten to be clearer.

We have attempted to improve the clarity of this section.

465-498: This paragraph currently reads as a collection of loosely connected ideas, which makes it difficult to follow. I recommend revising it into a clearer narrative. Consider breaking it into multiple paragraphs, with transition sentences to guide the reader through the logic.

Thank you for this suggestion. We have added further explanation of the ideas and observations made in this section.

502-503: The discussion of amino acid polymorphisms feels abrupt at this stage of the manuscript. If the authors intend to highlight these results, they should introduce earlier why amino acid polymorphisms are relevant to their study. This would provide better context and ensure the section integrates smoothly into the overall flow of the paper.

We modified this paragraph to provide a better explanation of our motivation. The introduction contains explanation and references supporting CENH3 as a key protein involved in centromere identity. We also provide CENH3 ChIP-seq data in panel D of Figures 2-6. As we are studying the effects of natural variation on recombination and distortion, we felt it was logical to inspect CENH3 polymorphisms present among the studied accessions. Our analysis clearly indicates that as CENH3 polymorphisms are rare amongst our sample of accessions, these are very unlikely to explain the observed variation in crossover frequency and segregation distortion.

504-505: The authors should explain why they are using A. lyrata. Why is variation at the N-terminal tail of CENH3 significant? What kind of variation are the authors referring to?

Arabidopsis lyrata is the closest species to *A. thaliana*, and the two species separated approximately 5-10 million years ago. CENH3 variation is known to be high between species, and low within species, within the Arabidopsis genus, and so this was included as a control comparison. We now provide references to prior work [PMID:17028323. PMID:21190064].

500-508: The conclusion presented in this paragraph is potentially valuable, but the connection between the evidence provided and the conclusion is unclear. I recommend clarifying how the data or arguments in the paragraph support this point so the reader can clearly follow the reasoning.

Thank you, we have modified the text here to make our motivation and reasoning clear.

527-540: This paragraph begins with large inversions and structural variants and then switches to QTLs. Can the authors add a smoother transition and add the relevance/connection.

This paragraph discusses the evidence for- *cis* and *trans*-modifying effects on crossover frequency in this study. We have modified the text to make this clear.

540-542: The transition into the discussion of meiotic drive in female meiosis feels abrupt. Since the study examines seeds rather than pollen, both female and male meiosis are being evaluated. Highlighting this connection could provide a reason for discussing this topic.

Thank you for this suggestion. We have modified this section to better place this material in context.

546: Can the authors expand on how this is similar in Arabidopsis?

We have added a more detailed description of ovule and megasporocyte development in Arabidopsis and how this might relate to female meiotic drive.

554: The authors should spell out "FISH".

We have spelt this out.

550-557: Can the authors explain the points of these two studies in relation to their study? How is the transmission ratio relevant to their case?

In this section of the discussion, we refer to two well-studied systems of female meiotic drive in *Mimulus* and mouse [PMID:25242031, PMID:28756949, PMID:15466426, PMID:19056989]. In these systems, the driving haplotype was found to correlate with a larger repeat array. In our case, we see the opposite, where distortion can occur in favour of the smaller centromere array in multiple cases. We have clarified these observations in the Discussion.
For the second study, are they trying to point out the centromere size or that their lab strains are not reliable? Please add some clarity.

Our centromere assemblies are complete, and so we conclude that these trends may vary between different distorting systems, which will be of interest to the field. We have added a sentence at the end of this section to make our conclusion clear.

566-587: For segregation distortion, the authors provide a good potential reason, but please further explain the "synapsis buckle".

We have expanded on this idea in the Discussion to make this concept more clear, and added a new Fig. S11 to illustrate this idea.

F1A: Please add a measure of diversity for each centromere.

This is not a straightforward request. We calculated pairwise pi, which is the most suitable metric for the non-array regions. Whereas, a comparison of *CEN178* repeat copy number or similarity is most appropriate for the core centromere regions. These measures of diversity are provided in Table S1.

F1B: Please add which strains are in which genetic group?

We have added accession name labels to this figure.

F2-6C: All the strains analyzed in this panel are different for each centromere and for each part of the figure. To try to figure out why, I examined the strains in part B that are significant to see if those are the same ones examined at in part C, but it doesn't hold. These are also not the same combination of strains examined at in part D, the subset to examine epigenetic organization. While the bar plot at the bottom in part C (CO frequency and segregation distortion) is clear and useful, the overall presentation of part C is confusing. The author needs a clearer way to convey this information.

The choice of centromeres to include in the sequence similarity dotplots in Figures 2 to 6 was based on those that showed significant changes to either crossover frequency, or segregation distortion, compared to the inbred control strain.

FS1: For the "DNA methylation" legend, the colors appear to be dark red, pink, light pink, instead of dark red, orange, and yellow. For the "features" legend, the "TEs" annotation appears to be orange, and not green. Updating the legend to reflect these colors would improve clarity.

Thank you for noticing these mistakes in the legend, which we have now corrected.

FS3: These plots are very small I can barely read them. There are parts A-E, but in the description, there are only parts A-D. The plots could be organized better to reduce the white space.

Thank you for spotting this. We have modified the legend to refer to part E.

957: Please state what TRASH is.

We have deleted mention of TRASH here, which is instead referenced in the Methods. This is a software used to map centromere satellite tandem repeats.

959: The correlation line looks more black than blue.

We have corrected the legend text.

FS4 & FS5: These figures seem out of order. Maybe FS4 can be switched so all the same graphs are next to each other.

The ordering of the Figures and Supplementals is based on the order in which the figures are called out in the text, as per journal instructions.

Reviewer #2 :

This is an interesting work that studies meiotic crossover around the centromeric region, as well as meiotic segregation distortion using fluorescent seed markers. Twelve Arabidopsis accessions, representing a broad range of ecogeographical diversity, and whose genomes and centromeric arrays were sequenced and fully assembled, were crossed to testers in the Col-0 background, one tester for each chromosome. Then, the authors attempted to correlate the crossover and segregation distortion to sequence or structural polymorphism, to

epigenetic patterns, or to CENH3 variants. So we are having in total 60 segregating populations of F2 seeds (12 parental lines x 5 testers in Col-0), and for each combination, the correlation with genetic and epigenetic parameters was analyzed.

While the reading was tough because of the abundance of details, overall, the work was well done. 12 Replicas were made for each hybrid, and a large number of seeds were counted, so that the statistics of crossover rate or segregation distortion measurements are solid.

Thank you for your positive comments and assessment.

I think that the results are important, and the discussion is fine. However, I would request that the authors revise their conclusions, in particular regarding the title, which is very vague and doesn't say much, and the abstracts, in a more direct/candid manner.

What I mean is that it seems that the authors are trying hard to say something positive about occasional trends, when in fact, it seems that each centromere in each cross is a different story, and that no clear conclusions can be drawn, and if there are some trends sometimes, they are weak, and they were not subjected to a statistical analysis. The author should embrace this complexity. In fact, I found that the strength of the work is in providing a strong insight into what DOES NOT explain crossover frequencies around the centromere or what DOES NOT explain segregation distortion. Ruling out possible mechanisms is very important for future work by others.

The main conclusion is that the size of the CENH3-enriched arrays, the sequence polymorphism of the region between the markers, and the patterns of methylation DID NOT correlate with crossover rates or with segregation distortion. Most effects were accession or centromere-specific. Moreover, there was NO correlation between centromere-proximal crossover and segregation distortion. These conclusions should be clear from the abstract and the title.

Our earlier response to the first reviewer is directly relevant to this point; so it is reproduced and modified slightly here.

First, as each FTL interval varies in physical size, we used crossovers per megabase (cM/Mb) as the response variable. We considered the model:

cM/Mb ~ chromosome * accession

Where ‘chromosome’ denotes the CTL interval analysed (*CEN1* to *CEN5*), and ‘accession’ denotes the hybrid background that was measured. Our model explained 85.01% of the variation in crossover frequency, where the interaction between chromosome and accession explained the greatest amount (53.49%), compared to chromosome (13.05%), and accession (7.27%) alone. This indicates that the influence of a specific centromere on crossover frequency is dependent on the accession crossed to. We tested additional models for crossover frequency that included the stepwise addition of the variables *CTL* interval length, gene density, transposon density, and *CEN178* density, but found these did not explain additional variation. Including segregation distortion data in the model explained a small amount of crossover variation (0.15% for red inheritance and 0.59% for green inheritance), consistent with observations made for individual centromeres. Genotype replicates were included as a random variable to control for experimental replication and were not found to contribute to the variance explained.

We took a similar approach to model genetic control of segregation distortion. For each *CTL* interval, we previously observed that the inheritance ratios of red and green fluorescence were highly correlated, and similar results were obtained modelling the red or green seed fluorescence data. We considered the model:

segregation distortion ~ chromosome * accession

The models explained 56.36% (green) and 53.22% (red) of the variation in distortion, where the interaction between chromosome and accession explained the greatest amount of variance

(green=35.19%, red=30.18%), compared to chromosome (green=1.99%, red=4.03%) and accession (green=20.58%, red=19.98%) alone. This indicates that, like for crossover frequency, the influence of a specific centromere is dependent on the accession crossed to.

In contrast to crossover frequency, accession had a stronger individual effect than chromosome. Including cM/Mb data as a variable explained a small amount of variation in distortion (green=2.24%, red=1.63%). In contrast to cM/Mb, including the annotation features FTL interval size, transposon density, gene density and *CEN178* density explained a greater amount of variation in distortion (green=69.45%, red=66.67%), potentially indicating a stronger contribution of *cis*-acting genetic variation on distortion compared to crossover frequency.

These analyses are now presented in the final section of the results, in Fig. 7, and Table S8. Fig S2, S3, and Table S1 also provide data and analysis across all five centromeres.

November 17, 2025
RE: GENETICS-2025-308714

Prof. Ian R. Henderson
University of Cambridge
Department of Plant Sciences
University of Cambridge
Cambridge, N/A CB2 3EA
United Kingdom

Dear Ian:

Congratulations, your manuscript titled "Natural variation modifies centromere-proximal meiotic crossover frequency and segregation distortion in Arabidopsis thaliana" is accepted for publication in GENETICS! Many thanks for submitting your research to the journal.

To Proceed to Publication:
1. Format your article according to GENETICS style: https://academic.oup.com/genetics/pages/author-guidelines

2. Ensure that you comply with data and community resource citation guidelines:
https://academic.oup.com/genetics/pages/author-guidelines#section-5-9-2

3. Upload your final files at https://genetics.msubmit.net

4. Add oupsupport@scipris.com and genetics.oup@novatechset.com (or the domains @scipris.com and @novatechset.com) to your email program's "safe senders" list. You will be contacted by both at various points during the production process.

Notes:
- Your currently-accepted manuscript (unedited, as submitted, reviewed, and accepted) will be published at GENETICS and deposited into PubMed as an Advance Access article. Notify sourcefiles@thegsajournals.org before signing your license if you do not wish to publish your article via Advance Access.

- We invite you to submit an original color figure related to your paper for consideration as cover art. Please email your submission to the editorial office or upload it with your final files. You can submit a small-sized image for evaluation, and if selected, the final image must be a TIFF file 2513px wide by 3263px high (8.375 by 10.875 inches; resolution of 600ppi). Please avoid graphs and small type.

- After files are sent to Oxford University Press we use SciPris to manage article licensing and payment. If you do not have a SciPris account, you will receive an email from no-reply@scipris.com to sign up to use Oxford University Press' author portal. After logging in, follow the online instructions to sign your license and arrange any payment due.

If you have any questions or encounter any problems while uploading your accepted manuscript files, please email the editorial office at sourcefiles@thegsajournals.org.

Sincerely,

James Birchler
Associate Editor
GENETICS

Approved by:
Amanda Larracuente
Senior Editor
GENETICS

Review comments (if applicable):

Reviewer #1 :

I am satisfied with the author's revisions.

Reviewer #2 :

I am satisfied by the revised version and the inclusion of the model that provides quantitative data on the various parameters studies (Chromosomes, genotypes, ..)
