# Supplementary material for: Natural variation modifies centromere-proximal meiotic crossover frequency and segregation distortion in Arabidopsis thaliana
Source: Genetics. 2025 Dec 8;233(4):iyaf264. doi: 10.1093/genetics/iyaf264 (PMC13439696; doi:10.1093/genetics/iyaf264)
Supplement: Supplementary file 2 [file iyaf264_supplementary_data.zip › Supplemental_Material_GENETICS-2025-308714.docx]

**Figure S1. Location of *CEN* FTL intervals relative to the Col-0 genome and genetic and epigenetic features.** For each chromosome in the Col-CEN assembly [(Naish *et al.* 2021)](https://paperpile.com/c/cT5TK8/yAkxW), we plotted the position of the FTL red-encoding (red triangles) and green-encoding (green circles) T-DNAs. Beneath we show density plots for (i) *CEN178* satellite repeats (green) and CENH3 ChIP-seq enrichment (purple), (ii) DNA methylation in CG (dark red), CHG (pink) and CHH (light pink) sequence contexts [(Naish *et al.* 2021; Wlodzimierz *et al.* 2023b)](https://paperpile.com/c/cT5TK8/yKF3C+yAkxW), (iii) gene density (blue), (iv) transposon density (orange), (v) histone H3K9me2 ChIP-seq enrichment (dark blue), and (vi) *5S* rDNA (grey).

**Figure S2. Correlation between FTL fluorescence ratios, crossover frequency and total seed counts. A.** On the left, a scatter plot of green:non-green, and red:non-red *CEN1* seed fluorescence ratios, for every genotype and replicate. On the right, a scatter plot of colour:non-colour (red and green plotted separately) and *CEN1* crossover frequency (centiMorgan), for every genotype and replicate. Where the correlation is significant, a blue line is printed showing the relationship. Correlation values are printed above, together with the *P* value. **B.** As for A., but showing data for *CEN2*. **C.** As for A., but showing data for *CEN3*. **D.** As for A., but showing data for *CEN4*. **E.** As for A., but showing data for *CEN5*. **F.** As for A., but plotting total seed counts for each replicate against either cM, or green:non-green, or red-non-red fluorescence ratios, for each of the centromeres.

**Figure S3. Correlation between FTL size, *CEN178* copies, crossover frequency, nucleotide diversity, gene and transposon density, and fluorescence inheritance ratios. A.** A scatter plot of *CEN178* repeat number and the physical size of the FTL interval, in each accession. Data from the *CEN1*, *CEN2, CEN3*, *CEN4,* and *CEN5*, are coloured separately. Where the correlation is significant, a black line is printed showing the relationship. Correlation values are printed above, together with the *P* value. **B.** As for A., but correlating FTL interval size with cM, or ratios of green:non-green, or red:non-red fluorescent seed counts. **C.** As for B., but comparing *CEN178* copy number. **D.** As for A, but pairwise nucleotide diversity (π) in FTL regions, with FTL width, *CEN178* copy number, centiMorgans, or ratios of green:non-green, or red:non-red fluorescent seed counts. **E.** As for A, but showing π compared to centiMorgan, and ratios of green:non-green, or red:non-red fluorescent seed. **F.** As for A, but comparing FTL interval size (Mb) against genes per megabase within the interval. **G.** As for A, but showing gene density compared to centiMorgan, and ratios of green:non-green, or red:non-red fluorescent seed. **H.** As for A, but comparing FTL interval size (Mb) against transposable element annotation per megabase within the interval. **I.** As for A, but showing transposable element density compared to centiMorgan, and ratios of green:non-green, or red:non-red fluorescent seed.

**Figure S4. Patterns of genetic variation on chromosome 1 between Col-0 and the twelve hybrid accessions, ordered by *CEN1* crossover frequency.** We used SyRI to map synteny between Col-CEN chromosome 1 (blue) and each of the accessions used to generate F_1_ FTL hybrids (orange) [(Goel *et al.* 2019)](https://paperpile.com/c/cT5TK8/U6Eo). Regions detected by SyRI as syntenic (grey), inverted (orange), translocated (green), and duplicated (blue), are shaded between the chromosomes. The SyRI comparison plots are arranged from top to bottom in order of ascending *CEN1* crossover frequency in the cognate F_1_ hybrid. To the right, we also list whether there was over- or under-transmission of the *CEN1* FTL chromosome compared to inbreds. Segregation distortion is measured as colour:non-colour (C:Non-C) ratios in the seed. At the top is a plot of Col-CEN chromosome 1 showing the positions of the FTL red-encoding (red triangle) and green-encoding (green triangle) T-DNAs, and the location of the centromeric *CEN178* satellite arrays (dark grey).

**Figure S5. Correlation between CENH3 enrichment area, FTL size, nucleotide diversity, *CEN178* copies, crossover frequency, and fluorescence inheritance ratios. A.** Scatter plots comparing the area of CENH3 ChIP-seq enrichment, against pairwise FTL nucleotide diversity (π), FTL interval size (Mb), *CEN178* satellite repeat copy number. Data points are coloured according to the accession. **B.** As for A, but comparing the area of CENH3 ChIP-seq enrichment against hybrid crossover frequency, green to non-green, and red to non-red fluorescence inheritance ratios.

**Figure S6. Patterns of genetic variation on chromosome 2 between Col-0 and the twelve hybrid accessions, ordered by *CEN2* crossover frequency.** We used SyRI to map synteny between Col-CEN chromosome 2 (blue) and each of the accessions used to generate F_1_ FTL hybrids (orange) [(Goel *et al.* 2019)](https://paperpile.com/c/cT5TK8/U6Eo). Regions detected by SyRI as syntenic (grey), inverted (orange), translocated (green), and duplicated (blue), are shaded between the chromosomes. The SyRI comparison plots are arranged from top to bottom in order of ascending *CEN2* crossover frequency in the cognate F_1_ hybrid. To the right, we also list whether there was over- or under-transmission of the *CEN2* FTL chromosome compared to inbreds. Segregation distortion is measured as colour:non-colour (C:Non-C) ratios in the seed. At the top is a plot of Col-CEN chromosome 2 showing the positions of the FTL red-encoding (red triangle) and green-encoding (green triangle) T-DNAs, and the location of the centromeric *CEN178* satellite arrays (dark grey).

**Figure S7. Patterns of genetic variation on chromosome 3 between Col-0 and the twelve hybrid accessions, ordered by *CEN3* crossover frequency.** We used SyRI to map synteny between Col-CEN chromosome 3 (blue) and each of the accessions used to generate F_1_ FTL hybrids (orange) [(Goel *et al.* 2019)](https://paperpile.com/c/cT5TK8/U6Eo). Regions detected by SyRI as syntenic (grey), inverted (orange), translocated (green), and duplicated (blue), are shaded between the chromosomes. The SyRI comparison plots are arranged from top to bottom in order of ascending *CEN3* crossover frequency in the cognate F_1_ hybrid. To the right, we also list whether there was over- or under-transmission of the *CEN3* FTL chromosome compared to inbreds. Segregation distortion is measured as colour:non-colour (C:Non-C) ratios in the seed. At the top is a plot of Col-CEN chromosome 3 showing the positions of the FTL red-encoding (red triangle) and green-encoding (green triangle) T-DNAs, and the location of the centromeric *CEN178* satellite arrays (dark grey).

**Figure S8. Patterns of genetic variation on chromosome 4 between Col-0 and the twelve hybrid accessions, ordered by *CEN4* crossover frequency.** We used SyRI to map synteny between Col-CEN chromosome 4 (blue) and each of the accessions used to generate F_1_ FTL hybrids (orange) [(Goel *et al.* 2019)](https://paperpile.com/c/cT5TK8/U6Eo). Regions detected by SyRI as syntenic (grey), inverted (orange), translocated (green), and duplicated (blue), are shaded between the chromosomes. The SyRI comparison plots are arranged from top to bottom in order of ascending *CEN3* crossover frequency in the cognate F_1_ hybrid. To the right, we also list whether there was over- or under-transmission of the *CEN4* FTL chromosome compared to inbreds. Segregation distortion is measured as colour:non-colour (C:Non-C) ratios in the seed. At the top is a plot of Col-CEN chromosome 4 showing the positions of the FTL red-encoding (red triangle) and green-encoding (green triangle) T-DNAs, and the location of the centromeric *CEN178* satellite arrays (dark grey).

**Figure S9. Patterns of genetic variation on chromosome 5 between Col-0 and the twelve hybrid accessions, ordered by *CEN5* crossover frequency.** We used SyRI to map synteny between Col-CEN chromosome 5 (blue) and each of the accessions used to generate F_1_ FTL hybrids (orange) [(Goel *et al.* 2019)](https://paperpile.com/c/cT5TK8/U6Eo). Regions detected by SyRI as syntenic (grey), inverted (orange), translocated (green), and duplicated (blue), are shaded between the chromosomes. The SyRI comparison plots are arranged from top to bottom in order of ascending *CEN5* crossover frequency in the cognate F_1_ hybrid. To the right, we also list whether there was over- or under-transmission of the *CEN5* FTL chromosome compared to inbreds. Segregation distortion is measured as colour:non-colour (C:Non-C) ratios in the seed. At the top is a plot of Col-CEN chromosome 5 showing the positions of the FTL red-encoding (red triangle) and green-encoding (green triangle) T-DNAs, and the location of the centromeric *CEN178* satellite arrays (dark grey).

**Figure S10. CENH3 amino acid variation present in the Arabidopsis accessions studied in comparison with *Arabidopsis lyrata*.** Protein sequences of the CENH3 coding region were extracted from the 13 studied Arabidopsis accessions, and *A. lyrata* (MN47), which contains two CENH3 paralogues (Al1G11082 and Al1G59290) [(Kawabe *et al.* 2006)](https://paperpile.com/c/cT5TK8/8Rl9). MAFFT (<https://github.com/GSLBiotech/mafft>) was used to align the protein sequences and snipit (<https://github.com/aineniamh/snipit>) was used for visualisation. Tick marks across the protein sequence represent polymorphisms compared to the reference Col-0 CENH3 sequence and above is shown the reference amino acid. The positions of the polymorphisms and the corresponding amino acid changes are shown at the top of the graph.

**Figure S11. Model for how centromere structural polymorphism may cause segregation distortion in Arabidopsis female meiosis. A.** Diagram representing the developing female ovule in Arabidopsis. A sub-epidermal cell becomes specified to enter meiosis and undergoes two cell divisions to produce a tetrad of haploid spores. Three of the megaspores undergo programmed cell death, and the surviving cell tends to be the innermost spore [(Webb and Gunning 1990; Bowman 2012)](https://paperpile.com/c/cT5TK8/PM7y+WXNH). The surviving megaspore then divides mitotically and differentiates into the female gametophyte, which contains the egg cell [(Webb and Gunning 1990; Bowman 2012)](https://paperpile.com/c/cT5TK8/PM7y+WXNH). **B.** In early prophase-I in the sub-epidermal cell that is specified to enter meiosis, the homologous chromosomes (red and blue) replicate and are held together by cohesin and the meiotic axis. During prophase I, the replicated chromosomes experience DNA double strand breaks (DSBs, stars) that initiate meiotic recombination. As cell division progresses, the replicated homologs pair to form a four-strand bivalent structure, and a subset of the DSBs are repaired using a homologous chromatid to form crossovers (black crosses). At this stage we propose there is an asymmetry in the spindle microtubules, either in number or arrangement (green). Due to centromere structural heterozygosity, we propose that the synapsed homologs form a buckle where the sequence is hemizygous. Therefore, the relative positioning of the CENH3 chromatin (ovals) is offset between the homologs in the bivalent. In this case, this synapse-buckle and CENH3-offset cause a biased association of the purple centromeres with the lower spindle. The chromosomes then segregate through meiosis-I and meiosis-II, and because of the initial bias, the blue centromere has a higher chance of segregating into the surviving female spore that escapes programmed cell death and differentiates into the female gametophyte and egg cell.

**Table S1. FTL interval size, annotation, crossover frequency, and segregation distortion for each centromere and hybrid.** For each FTL interval (*CEN1, CEN2*, *CEN3*, *CEN4* and *CEN5*), the accession name crossed to is listed, together with the associated genome assembly file name and which SNP PCA genetic similarity group the accession belongs to (Eurasian, Iberian relict, or Non-Iberian relict) [(Wlodzimierz *et al.* 2023b)](https://paperpile.com/c/cT5TK8/yKF3C). The row listing the Col-0 accession represents the inbred control line, which the twelve hybrids are crossed to. FTL T-DNA flanking sequences from the Col-CEN assembly were mapped to each genome assembly and used to calculate FTL start and end coordinates, and the width of the interval in each accession. For each genotype, the mean crossover frequency (centroMorgan, cM), and red:non-red-fluorescent (R:NR), and green:non-green-fluorescent (G:NG) is listed. The count data used for these calculations are provided in **Tables S3-S7**. Also listed are the number of *CEN178* satellite repeats annotated on this chromosome by TRASH [(Wlodzimierz *et al.* 2023a)](https://paperpile.com/c/cT5TK8/Nks5), and the Jaccard similarity of the *CEN178* sequences present in each accession in each centromere with those in the Col-CEN assembly. Pairwise nucleotide diversity (π) relative to Col-0 was calculated for each accession using SNPs in the FTL T-DNA intervals on each chromosome. For each FTL interval in Col-0, we also add the count of genes, as well as the % of the interval occupied by gene annotation, as well as the same values for TEs, and *5S* rDNA. For each accession where we calculated the width of zones of CENH3 ChIP-seq enrichment (bp), as well as the number of discrete zones of enrichment. Gene and transposable element density are also listed for each FTL interval in each accession.

**Table S2. *Arabidopsis thaliana* accessions analysed in this study.** Accession name is listed, together with chromosome arm SNP PCA genetic similarity group (Eurasian, Iberian relict or Non-Iberian relict), and the country, latitude, and longitude of the collection location. We provide references for genome assemblies of accessions used for analysis.

**Table S3. Fluorescent FTL seed count data for *CEN1*.** The table lists fluorescent seed count data from replicate hybrid F_1_ plants, where the accession listed was crossed to Col-0 *CEN1* lines. Counts of green-alone (*N_G_*), red-alone (*N_R_*), both red and green fluorescent, and non-fluorescent seed, as well as the total number of seeds are listed, for each replicate. For each replicate, we also present crossover frequency (cM, centiMorgan), as well as the ratio of green to non-green fluorescent seed (G:NG), the ratio of red to non-red fluorescent seed (R:NR), and the ratio of green to red seed (G:R). The final column lists if genotypes were inbred or hybrid.

**Table S4. Fluorescent FTL seed count data for *CEN2*.** The table lists fluorescent seed count data from replicate hybrid F_1_ plants, where the accession listed was crossed to Col-0 *CEN2* lines. Counts of green-alone (*N_G_*), red-alone (*N_R_*), both red and green fluorescent, and non-fluorescent seed, as well as the total number of seeds are listed, for each replicate. For each replicate, we also present crossover frequency (cM, centiMorgan), as well as the ratio of green to non-green fluorescent seed (G:NG), the ratio of red to non-red fluorescent seed (R:NR), and the ratio of green to red seed (G:R). The final column lists if genotypes were inbred or hybrid.

**Table S5. Fluorescent FTL seed count data for *CEN3*.** The table lists fluorescent seed count data from replicate hybrid F_1_ plants, where the accession listed was crossed to Col-0 *CEN3* lines. Counts of green-alone (*N_G_*), red-alone (*N_R_*), both red and green fluorescent, and non-fluorescent seed, as well as the total number of seeds are listed, for each replicate. For each replicate, we also present crossover frequency (cM, centiMorgan), as well as the ratio of green to non-green fluorescent seed (G:NG), the ratio of red to non-red fluorescent seed (R:NR), and the ratio of green to red seed (G:R). The final column lists if genotypes were inbred or hybrid.

**Table S6. Fluorescent FTL seed count data for *CEN4*.** The table lists fluorescent seed count data from replicate hybrid F_1_ plants, where the accession listed was crossed to Col-0 *CEN4* lines. Counts of green-alone (*N_G_*), red-alone (*N_R_*), both red and green fluorescent, and non-fluorescent seed, as well as the total number of seeds are listed, for each replicate. For each replicate, we also present crossover frequency (cM, centiMorgan), as well as the ratio of green to non-green fluorescent seed (G:NG), the ratio of red to non-red fluorescent seed (R:NR), and the ratio of green to red seed (G:R). The final column lists if genotypes were inbred or hybrid.

**Table S7. Fluorescent FTL seed count data for *CEN5*.** The table lists fluorescent seed count data from replicate hybrid F_1_ plants, where the accession listed was crossed to Col-0 *CEN5* lines. Counts of green-alone (*N_G_*), red-alone (*N_R_*), both red and green fluorescent, and non-fluorescent seed, as well as the total number of seeds are listed, for each replicate. For each replicate, we also present crossover frequency (cM, centiMorgan), as well as the ratio of green to non-green fluorescent seed (G:NG), the ratio of red to non-red fluorescent seed (R:NR), and the ratio of green to red seed (G:R). The final column lists if genotypes were inbred or hybrid.

**Table S8. Summary of linear models for crossover frequency and segregation distortion.** The table lists the linear models tested to explain variation in crossover frequency (cM/Mb), or red and green fluorescence ratios (RNonR and GNonG), as the response variables. The % of variation in the response variable for each term is provided, in addition to the total *R^2^* value for the model.
